# Spatiotemporal distribution of microplastics in Miri coastal area, NW Borneo: inference from a periodical observation

**DOI:** 10.1007/s11356-023-29582-7

**Published:** 2023-09-09

**Authors:** Anshuman Mishra, Prasanna Mohan Viswanathan, Nagarajan Ramasamy, Saravanan Panchatcharam, Chidambaram Sabarathinam

**Affiliations:** 1 Department of Applied Sciences, Faculty of Engineering and Science, Curtin University, Malaysia, CDT 250, 98009 Miri, Sarawak Malaysia; 2 Department of Geology, University of Madras (Guindy Campus), Chennai, 600025 India; 3 Kuwait Institute for Scientific Research, Kuwait City, Kuwait

**Keywords:** MPs, Sediment, Fibre, Polymer, Miri coast

## Abstract

The current study aims to investigate the spatiotemporal distribution of microplastics (MPs) in the Miri coast, targeting their occurrences, characterisation, and potential sources. For a periodical study, coastal sediments were collected from three different time intervals (monsoon, post-monsoon, and post-COVID) and subjected to stereomicroscope, ATR-FTIR, and SEM-EDX analyses. These results show a significant increase of MPs in post-COVID samples by approximately 218% and 148% comparatively with monsoon and post-monsoon samples, respectively. The highest concentration of MPs was detected near the river mouths and industrial areas where the waste discharge rate and anthropogenic activities dominate. Fibre-type MPs are the most abundant, with an average of nearly 64%, followed by fragments, films, microbeads, and foams. The most dominant polymer types were polytetrafluoroethylene (PTFE), polyethylene (PE), polypropylene (PP), polystyrene (PS), and polyester (PET). Overall, the current study shows a better understanding of MPs occurrence and potential sources in the Miri coastal area.

**Supplementary Information:**

The online version contains supplementary material available at 10.1007/s11356-023-29582-7.

## Introduction

In the twenty-first century, with the growth of civilisation, plastic pollution is also growing as a significant global concern, adversely affecting the surrounding ecosystems and human health. Plastics are manufactured from synthetic organic polymers, usually formed through the polymerisation of monomers created from fossil fuels and manufactured from polymer chains (Ivleva et al. 2017; Zhang et al. 2021). Plastics have different identical properties like durability, handy to use, lightweight, attractive designs, and low cost, which are popularly used all over the globe. Because of its widespread use, the current age is also known as the plastic age (Pan et al. 2020). Due to the widespread use of plastic products, there is a remarkable growth in non-biodegradable pollutants throughout several environments. From an estimation, the global production of plastics in 1950 was approximately 2.3 million tons, reaching 322 million tons at the end of 2015. According to the projected data, it may reach 33 billion tons in 2050 (Dümichen et al. 2017). Freshwater ecosystems (including rivers, streams, lakes, and ponds) are more prone to MPs pollution as they directly take their sediments from terrestrial environments either through inadequate waste disposal or loss from a landfill or transported through wind/surface run-off. MPs in the marine environment are widely identified as oceans/seas and are the ultimate ends of any aquatic system. There are several sources of MPs, but those MPs found in the marine environment are mainly from anyone, like mismanagement of waste treatment plants, directly from rivers, fishing activities, illegal dumping, or accidental cargo vessels (Horton and Dixon 2018). Among the land-based sources, municipal solid wastes are the primary concern in developing countries. Nearly 88% of municipal wastes are disposed in open dumps or landfills, producing plastic litter through various environmental influences (Pariatamby et al. 2020). MPs are also identified at different depths in the marine environment based on their density. Some denser particles are acrylic, polymerising vinyl chloride (PVC), and polyamide (PA), which are expected to sink and are found with marine sediments. At the same time, some others are quite lighter, like polyethylene (PE), polypropylene (PP), and polystyrene (PS), which are found in the floating stage (Smith et al. 2018).

MPs are commonly classified in size ranges, colours, and shapes (Kelly et al. 2021; Ramkumar et al. 2022). As per the size classification distribution, MPs generated from plastics having a length of more than 1 μm and less than 5000 μm, where the longest dimension of the particle measures (Lee et al. 2021; Rodríguez-Narvaez et al. 2021; Ramkumar et al. 2022). These diversifications of MPs occur due to the degradation of larger plastics. Once they are exposed to any environment, the leaching is more, and simultaneously, they bioaccumulate in marine organisms (Teuten et al., 2009). A current study reveals that the high global production of plastics, their consumption, and low degradation rate create a higher concentration of MPs in marine and freshwater environments (Bergmann et al. 2017). Several studies provide evidence regarding their abundance in the water column, sediments, and organisms through oceanic circulation and coastal currents (Wright et al. 2013). They are the dominant pollutants in several ecosystems like surface water (Lusher et al. 2016), estuaries (Firdaus et al. 2020), deep-sea sediments (Martin et al. 2017), coastal regions (Jung et al. 2021), groundwater (Koelmans et al. 2019), freshwater (Peng et al. 2017), ice caps in polar regions (Munari et al., 2017), and air (Chen et al. 2020). In the twenty-first century, the enormous growth of MPs pollution in the coastal environments is a global concern due to the discharge of plastic waste from terrestrial and marine sources (Karthik et al. 2018). In addition to the pre-existing plastic pollution, the COVID-19 pandemic played a crucial role in rising microplastic pollution around the globe through the degradation of several improper disposal of medical wastes (Aragaw 2020; Ebekozien et al. 2023; Hasan et al. 2023). Due to its high effectiveness and low cost in stopping the spread of the virus, personal protective equipment (PPE) has seen a significant surge in demand and use as a result of the COVID-19 pandemic. Remarkably, the use of gloves and face masks has significantly increased (Prata et al. 2020; Ammendolia et al. 2021), which leads to PPE might be an especially prevalent type of garbage in the ocean (Ardusso et al. 2021). Several researchers have demonstrated that the rise of plastic pollution in the coastal environments is because of anthropogenic activities, which worsened during the COVID-19 pandemic due to incorrect handling of medical waste (Dioses-Salinas et al. 2020; Cordova et al. 2021). PPEs were produced and utilised in large quantities throughout the COVID-19 pandemic, but their handling was challenged by the abrupt increase that has recently resulted in a worldwide issue (Dioses-Salinas et al. 2020; Chowdhury et al. 2021; De-la-Torre et al. 2021; Mohammadi et al. 2022).

Furthermore, the United Nations Sustainable Development (UN-SDGs) related its seven targets out of the 17 SDGs imposed in 2015 by examining the exponential development in the production of plastic trash and its influence on the surrounding ecosystems (Hossain et al. 2021). The seven UN-SDGs that are related to plastic pollution and its adverse effects on surrounding ecosystems are goal 3 (good health and well-being), goal 6 (clean water and sanitation), goal 11 (sustainable cities and communities), goal 12 (responsible consumption and production), goal 13 (climate action), goal 14 (life below water), and goal 15 (life on land) (Hossain et al. 2021; Khant and Kim 2022). Therefore, it is crucial to research MPs’ effects on the surrounding ecosystems.

However, several studies have been carried out on MPs pollution, distribution, and their impacts on ecosystems worldwide, but in the case of East Malaysia, it is rare. Only three studies have been conducted in the Miri regions, such as in the Miri River (Liong et al. 2021), Baram River (Choong et al. 2021b), and the Miri coast (anak Alexander Tampang and Mohan Viswanathan 2022). Even though these studies explained the occurrence, distribution, and characteristics of MPs, no studies have revealed the seasonal variation of MPs in this coastal environment. Hence, the main objectives of the current study are to do the characterisation study (i.e. size, shape, colour, and polymer type) of MPs. The study also reveals the potential source materials of MPs through morphometric analysis and the spatial-temporal distribution of MPs by comparing pre- and post-COVID occurrences of MPs on the Miri coast.

## Study area

Miri is a coastal city in the north-eastern part of Sarawak, Malaysia, with a population of more than 300 thousand, as per 2020 data. It is one of the most populated cities in the state. The city has significant tourist places to some world-famous UNESCO World Heritage sites, like Loagan Bunut National Park, Gunung Mulu National Park, Miri-Sibuti Coral Reef National Park, and Lambir Hills National Park. The current study area comes under the Miri region, starting from latitude 3°59′20″ N and longitude 113°43′10″ E to latitude 4°35′36.13″ N and longitude 113°58′21.54″ E. The South China Sea forms the entire western border of Miri City. The study was conducted on the Miri coastal and estuaries as these transitional zones have higher tidal action and sedimentation with densely populated regions. Tides also play an essential role in controlling the sedimentation rate due to the interaction between sea and coast in the study area.

According to an earlier study, both semidiurnal (two high tides and two low tides every day) and mixed tides (a combination of diurnal and semidiurnal tides) can be observed in this region (Rashidi et al. 2021). There are mainly two monsoonal climates observed in Miri: the SW monsoon from late May to September and the NE monsoon from November to March. October and April are known as two inter-monsoon seasons or transition periods (Billah et al. 2016; anak Alexander Tampang and Mohan Viswanathan 2022). The SW monsoon is mainly dry, so it is demarcated as pre-monsoon in this region. However, a lesser amount of rainfall is also noticed during these periods, so it is considered post-monsoon for the first 3 months (Anandkumar et al. 2022). Being a coastal city, Miri is enriched with petroleum basins, forests, caves, and major industries like timber processing, petroleum industries, Senadin Industrial area, Piasau Industrial Estate, Kuala Baram Estate, palm oil production industries, plywood, plastic, port, and shipyard industries (Choong et al. 2021a, b). An enormous quantity of sediments and water carrying several pollutants are discharged into the South China Sea by the major rivers of this region like Baram, Sibuti, and Miri (Nagarajan et al. 2013). However, some Sarawak beaches suffer severe pollution from natural causes or anthropogenic activities. Plastic bags and bottles are widely used due to their convenience to carry and dispose of. Improper waste disposal in this coastal environment by tourists ends up in the rivers and oceans (Wong et al. 2020). Based on an earlier study, it has been found that, in Miri, most municipal solid wastes are generated from food, cardboard, packaging, and paper, which accounts for about 0.83 kg per person in a day. Paper and plastic bags are mainly reusable, and Sibu has the most significant recycling rates (54.7%), followed by Miri (43%) and Kuching (42.7%) in East Malaysia. Miri has the most efficient solid waste collection (70.7%) (Pengurusan Sisa Pepejal Perbandaran Malaysia et al. 2018). Although there are a few concern agencies have been working on the collection of municipal solid waste and making public awareness of the proper waste disposal treatment, plastic pollution has been enormously increased on the Miri coast, which may be due to the negligence or several discharges of rivers and drainages on the coast of South China Sea. The sampling sites were chosen along the Miri coast based on the physiography, recreational centre, and port activities.

## Materials and methods

### Materials

Based on the standard protocols used in earlier studies for MPs analysis, all the analytical grade chemicals and apparatus are used in the current study. The chemicals used for the MPs analysis in this study are zinc chloride (Merck brand), hydrogen peroxide (30%, Merck), Whatman filter paper (0.45 μm pore size and 47 mm diameter), as well as distilled water was used for preparing all the solution.

### Sampling and quality control assurance

**Fig. 1:**
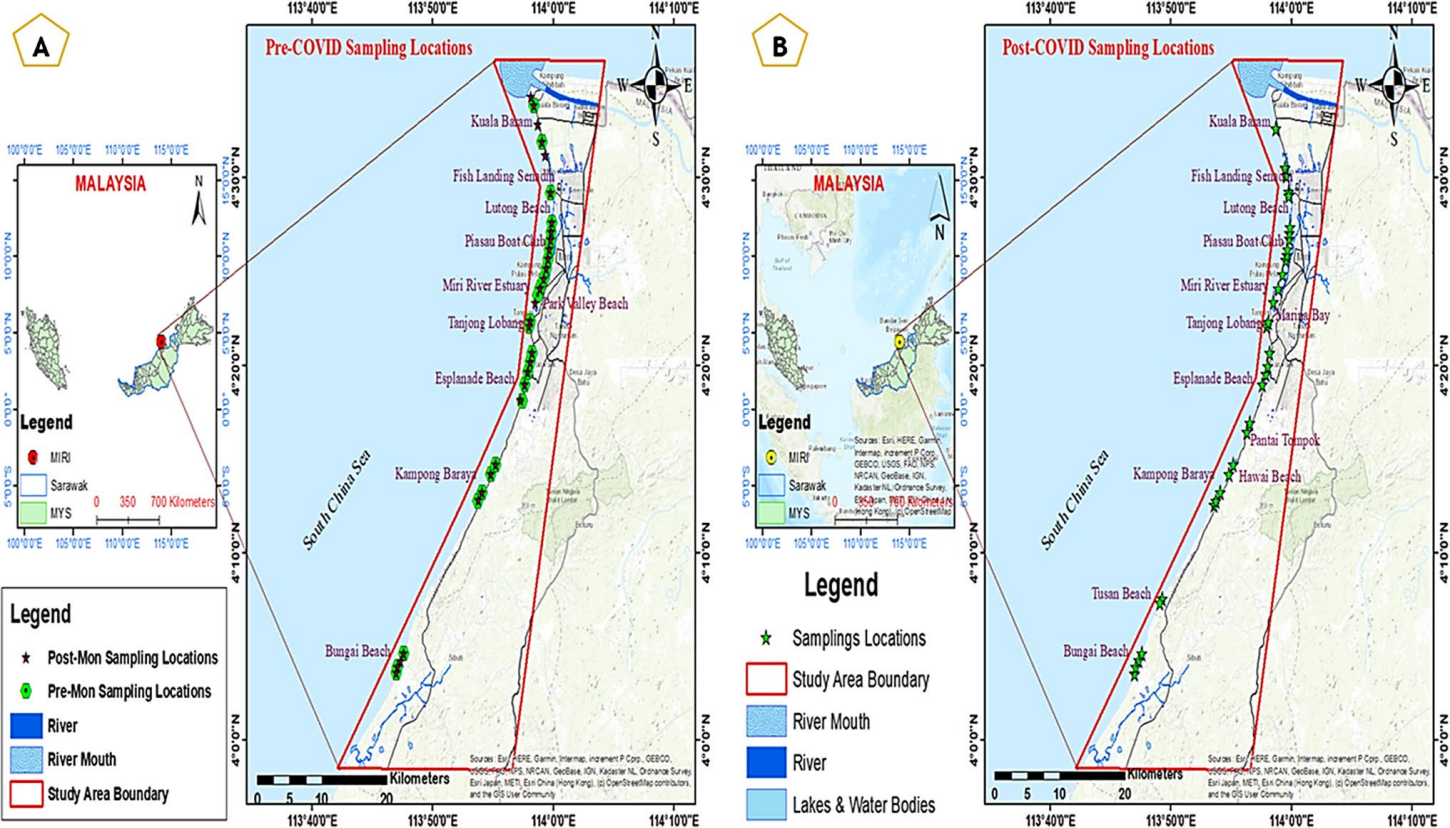
Study area representing the sampling locations map—(**A)** pre-COVID and (**B)** post-COVID

Sampling sites have been chosen based on population density, land use practice, seasonal changes, and the amount of discharge of water/sediments from significant rivers in the study area. The sediment samples were collected from selected locations along the Miri coast. These were solid or liquid waste discharge points to the marine environment, recreational activity centres, construction sites, and port activities sites (Fig. 1). Samples were collected in two different seasons to determine the seasonal impacts of MPs distribution. Hence, the first sampling was carried out during December 2013 as the monsoon season sample, and the second was done during June 2014 as the post-monsoon season sample. In addition, a recent sampling was carried out in July 2022, which fortunately helps to demonstrate a comparative study of the occurrence and distribution of MPs between the earlier samples (considered as pre-COVID) and current samples (post-COVID). However, there was a sampling gap of nearly 6 to 8 years. There was no significant variation in the land use and land cover (LULC) of the region for the studied periods. The comparison demonstrated that no significant changes had occurred during this period, except for a few establishments in forested areas (detailed of the LULC comparisons demonstrated in the supplementary material, i.e. Supplementary Table 1, Supplementary Figs. 1 and 2). Apart from this, the pre-COVID samples were analysed to demonstrate the seasonal variation of MPs abundancy in the regions and simultaneously the post-COVID samples were analysed by keeping the earlier time frame as the baseline of the study. Along with this, the MCOs during the COVID had also slowed down industrial growth, but a drastic increase in the domestic and urban solid waste in the region.

The coastal sediment samples were collected directly from the surface (i.e. within the surface to 5 cm) by a stainless-steel shovel. This was carried out because the surface samples were fresh. In contrast, samples beneath a few centimetres from the surface might be intermixed or decomposed, which will not be helpful for polymer identification (anak Alexander Tampang and Mohan Viswanathan 2022). From each coastal sampling site, 2 kg of sediment samples was collected and kept within aluminium foil mounting inside zipper bags. Samples were collected from 26 sampling sites from 10 beaches during the monsoon season and 30 locations from 11 beaches during post-monsoon, considered pre-COVID samplings. At the same time, the third or current sampling is taken into account as the post-COVID sampling carried out from 33 locations of 10 beaches. During sampling, the sampling intervals were always maintained (within 1–2 km) to get consistent data about the study area. The same research group conducted another study by taking 24 sediment samples from eight locations along the Miri coast in 2021, considered during the pandemic study (anak Alexander Tampang and Mohan Viswanathan 2022). The results of this study were compared with the outcomes of the each time frame of the current study, and for the series evaluation of MPs occurrence seasonal and pre-, during and post-COVID data have also been used. 

For the elimination of possible contamination, quality assurance was measured in both pre-sampling and post-sampling methods. In the initial phase, all samplers restricted themselves to garments to minimise the risk of contamination. Gloves were consistently used during the sampling process and subsequent analysis to uphold the quality and reliability of the samples. These standardised protocols were implemented to safeguard against external contamination and ensure the integrity and accuracy of the collected samples (Zhu et al. 2021). All the sample bottles and glass containers were washed thoroughly three times with distilled water and dried for at least 30 minutes before keeping the samples there. Three blank samples were also analysed with purified water to monitor the contamination level from the experiment work (Xu et al. 2020). Non-polymer research coats, caps, and gloves were used by the researcher to avoid contamination. All the apparatus and materials used for analysis were rinsed with distilled water three times. After the dissection of the samples, they were covered with aluminium foil to avoid contamination by airborne MPs (Zhu et al. 2021). For data reliability, all the filter papers used for different locations of samples were numbered. After filtering, all the filter papers were kept within glass Petri dishes individually with proper numbering and then dried. The flow chart of the research methodology has been added to the supplementary material (Supplementary Fig. 3).

### Extraction and analysis of microplastics

#### Microplastics extraction

For the extraction of MPs from the beach sediments, these sediments were dried at 60 °C for 48 hours using the oven (Brand-Binder Model: ED 260) in the laboratory. After drying the sediment samples, these were sieved by a sieving pan of aperture size 5 mm to separate the coarser sediments and organic matter having a larger diameter than 5 mm. The coning and quartering method was adopted for collecting sediments, followed by 30 g of weighed sediments collected from these. To remove organic matter from these sediments, they were treated with hydrogen peroxide (H_2_O_2_—30%) using a 250-ml glass beaker for 24 hours. The density separation method was adopted to extract MPs from the treated sediments. The zinc chloride (ZnCl_2_) solution was prepared using a magnetic stirrer and kept undisturbed for 24 hours. After that, this supersaturated solution was added to the pre-treated sediments and kept overnight to settle the denser particles (>1.6 g/cc). The MPs were separated by filtration using 0.45 μm pore size Whatman cellulose nitrate membrane filter paper (having 47 mm diameter) in the vacuum pump. After filtration, the filter paper was transferred to a glass Petri dish and left air-dried for 2 to 3 days or dried at 40 °C for 3 hours. The filter paper was kept for further quantification and characterisation study of MPs (Rodrigues et al. 2020; anak Alexander Tampang and Mohan Viswanathan 2022).

#### Analysis of microplastics and polymer identification

The dried filter papers were analysed to extract and classify MPs under the stereomicroscope (model—Nikon SMZ745T). MPs were classified based on their colours, sizes, and shapes (Kelly et al. 2021; Ramkumar et al. 2022). More than eight types of MPs based on colours (e.g. red, green, blue, white, yellow, black, violet, transparent) and six types (e.g. fibre, fragment, pellet/sphere, sheet, foam, film) MPs based on shapes were identified and quantified separately for each sampling location. As per the size classification distribution, MPs generated from plastics having a length of more than 1 μm and less than 5000 μm, where the longest dimension of the particle (Lee et al. 2021; Rodríguez-Narvaez et al. 2021; Ramkumar et al. 2022). The morphology and elemental compositions present in the MPs extracted from the sediments were determined by a scanning electron microscope (SEM—Thermo Fisher Scientific, Origin—USA) equipped with energy dispersive X-ray (EDX). This was used to determine the microphotographs of the MPs particles to know the surface structure and the possible mode of degradation. The voltage of the EDX was decided by considering the condition and resolutions of the polymers on the stubs, i.e. it varies between 5 and 20 kV.

The extracted MPs were analysed through Raman spectroscopy and Fourier transform infrared (FTIR) spectroscopy to identify different polymers based on their spectral range (Vetrimurugan et al. 2020). For the pre-COVID samples, a total of 15 samples were selected (representing one from every shape and each colour) to do Raman spectroscopy analysis, which was done by Horiba Xplora Plus Raman microscope spectrophotometer (origin: Bangalore, India) equipped with a binocular microscope powered by LabSpec 6 software. For spectrum capture and sample detection, an x50_VIS_LWD objective was used. Raman scattering was stimulated by using a 532-nm red laser diode with a laser power of 100 mW. Scanning was done with an acquisition time of 3 to 5 s (depending upon the condition of the samples) in the 50 to 4000 cm^−1^ spectral range. With a hole size of 100 μm and a slit size of 100 μm, a diffraction grating with 1800 grooves/mm was utilised. The peaks were matched against the KnowItAll Raman Spectral Library Database collection. For the post-COVID samples, a total of 20 extracted MPs, five common plastics (for standard comparisons), and five face mask samples were analysed by Agilent Cary 360-FTIR equipped with an in-built diamond attenuated total reflection (ATR) having a transmittance spectral range of 650 to 4000 cm^−1^. For the generation of the MPs spectrum, each particle was pressed onto the diamond crystal by the swivel pressure tower and operated through transmittance mode at 4 cm^−1^ resolution by the MicroLab software. After this, the peaks were generated by the wave number (cm^−1^) versus the transmittance spectrum. The polymer types were evaluated by matching the peaks with the KnowItAll Spectral Library and Bio-Rad Library, where the match score counted out of 100 (Lee et al. 2021; anak Alexander Tampang and Mohan Viswanathan 2022).

#### Statistical analysis

Statistical Package for Social Studies (SPSS) version 17.0 (IBM Corp., USA) was used to analyse the results obtained from the characterisation study. In order to investigate the pathogenic aspects of MPs, principal component analysis (PCA) and factor analysis (FA) were conducted (Li et al. 2020). Statistical analysis was used to evaluate the variations between the several size ranges, the quantification and the shapes of MPs, which also helped demonstrate the MPs’ prevalence (Paul et al. 2019; Song et al. 2021).

## Results

### Abundance of microplastics

The abundance and the concentration of MPs in the proposed study area are determined in terms of particles per 30 g beach sediments, with percentages. Out of the 26 sampling sites during the monsoon season, Kuala Baram (KB 01) is listed as the highest abundance of MPs, i.e. 21 particles/30 g with a concentration of 6.77% (Table 1), while the least abundance of MPs is detected in PBC 01 (Piasau Boat Club), with an abundance of 5 particles/30 g and a concentration of 1.61% only. Fibre type of MPs is the most abundant type with a concentration of 68.39%, while the sheet is the least abundant type of MPs with a concentration of 1.61%. In the same context, during the post-monsoon season, the highest number of MPs is detected in FS(II) 01 (Fish Landing Senadin), with a total of 33 particles/30 g with a concentration of 7.21% (Table 2), whereas the least abundance of MPs is detected from six sampling sites named KBR(II) 02, ESP(II) 02, ESP(II) 03, TB(II) 02, MR(II) 02, and MR(II) 03 with an equal number of abundance as well as concentration, i.e. nine particles/30 g and 1.97%, respectively. Furthermore, fibres are the most abundant MPs, with a concentration of 70.74%, and foam is the least abundant type, with a concentration of 1.97%.

**Table 1:** Occurrence and shapes of MPs in each 30 g of beach sediment (monsoon)

Sample ID	Shapes of MPs						Total particles/30 g	Total in %
	Fibre	Film	Fragment	Foam	Sheet	Microbeads		
B 01	12	2	2	1	0	2	19	6.13
B 02	7	1	1	0	1	0	10	3.23
B 03	8	1	1	0	0	3	13	4.19
KBR 01	10	1	2	1	0	2	16	5.16
KBR 02	6	0	0	0	0	0	6	1.94
KBR 03	11	0	2	0	0	4	17	5.48
HB 01	5	0	2	0	0	0	7	2.26
ESP 01	14	0	0	0	0	0	14	4.52
ESP 02	11	1	0	0	0	0	12	3.87
ESP 03	6	1	0	0	0	2	9	2.90
ESP 04	6	0	1	0	0	2	9	2.90
ESP 05	7	0	1	0	0	0	8	2.58
TB 01	5	0	1	0	0	3	9	2.90
TB 02	12	1	2	0	0	5	20	6.45
MR (PRABA) 01	6	0	1	2	0	1	10	3.23
MR 01	9	0	1	2	0	0	12	3.87
MR 02	10	0	1	0	0	0	11	3.55
MR 03	2	0	0	2	0	2	6	1.94
MR 04	8	2	3	1	2	4	20	6.45
PBC 01	2	0	0	1	1	1	5	1.61
PBC 02	4	0	1	0	0	2	7	2.26
Lu 01	10	0	2	0	0	0	12	3.87
Lu 02	11	1	1	0	0	4	17	5.48
Lu 03	7	0	0	0	0	3	10	3.23
FS 01	8	0	2	0	0	0	10	3.23
KB 01	15	1	1	0	1	3	21	6.77
Total	212	12	28	10	5	43	310	100.00
In %	68.39	3.87	9.03	3.23	1.61	13.87	100.00

**Table 2:** Occurrence and shapes of MPs in each 30 g of beach sediments (post-monsoon)

Sample ID	Shapes of MPs						Total particles/30 g	Total in %
	Fibre	Film	Fragment	Foam	Sheet	Microbeads		
B (II) 01	8	1	2	0	0	2	13	2.84
B (II) 02	10	0	1	0	0	2	13	2.84
B (II) 03	11	0	1	0	0	3	15	3.28
B (II) 04	9	1	0	0	1	2	13	2.84
KBR (II) 01	11	1	2	1	0	2	17	3.71
KBR (II) 02	8	0	1	0	0	0	9	1.97
KBR (II) 03	10	0	1	1	1	4	17	3.71
HB (II) 01	11	0	1	1	1	3	17	3.71
ESP (II) 01	12	2	0	0	0	0	14	3.06
ESP (II) 02	6	0	2	1	0	0	9	1.97
ESP (II) 03	8	0	1	0	0	0	9	1.97
ESP (II) 04	15	2	2	0	2	0	21	4.59
ESP (II) 05	12	0	0	1	1	0	14	3.06
TB(II) 01	11	1	2	1	0	4	19	4.15
TB(II) 02	8	0	0	0	1	0	9	1.97
PVB (II) 01	12	0	2	0	0	3	17	3.71
MR (II) 01	10	0	0	0	0	2	12	2.62
MR (II) 02	6	0	1	0	0	2	9	1.97
MR (II) 03	9	0	0	0	0	0	9	1.97
MR (II) 04	12	1	1	0	0	3	17	3.71
PBC (II) 01	10	1	2	0	1	0	14	3.06
PBC (II) 02	8	0	0	0	1	3	12	2.62
Lu (II) 01	16	1	2	1	1	3	24	5.24
Lu (II) 02	14	0	2	0	1	4	21	4.59
Lu (II) 03	11	1	2	0	0	4	18	3.93
FS(II) 01	10	0	0	0	0	2	12	2.62
FS(II) 02	20	2	3	1	2	5	33	7.21
KB (II) 01	12	0	2	0	0	3	17	3.71
KB (II) 02	14	1	2	1	0	3	21	4.59
KB (II) 03	10	0	1	0	0	2	13	2.84
Total	324	15	36	9	13	61	458	100.00
In %	70.74	3.28	7.86	1.97	2.84	13.32	100.00	

In addition to the above spatiotemporal observation of the MPs that occurred in the proposed study area, a similar study was conducted by considering the COVID-19 pandemic condition to establish a correlation between the pre- and post-COVID occurrence of MPs in this environment. For post-COVID samples, MR 01 (Miri River Estuary) has the highest number of MPs, i.e. 43 particles/30 g with a concentration of 6.37% (Table 3). On the other hand, HB (Hawai Beach) has the least abundance of MPs, i.e. 16 particles/30 g, with a concentration of 2.37%. Apart from this, fibres are the most abundant, and pellets are the least abundant MPs type observed in the region, having a concentration of 63.26% and 1.04% of MPs, respectively.

**Table 3:** Occurrence and shapes of MPs in each 30 g of beach sediments (post-COVID)

Sample ID	Shapes of MPs							Total particles/30 g	Total in %
	Fibre	Film	Fragment	Foam	Pellet	Sheet	Microbeads		
B 01	18	1	3	2	0	1	0	25	3.70
B 02	20	1	4	4	2	0	5	36	5.33
B 03	21	2	5	8	0	2	0	38	5.63
B 04	12	1	4	3	1	1	0	22	3.26
TuB 01	18	0	3	0	0	1	4	26	3.85
TH	28	2	1	3	0	1	4	39	5.78
KBR 01	14	0	2	5	0	0	0	21	3.11
KBR 02	17	0	2	4	0	0	0	23	3.41
KBR 03	15	0	3	4	0	0	2	24	3.56
HB	11	0	2	3	0	0	0	16	2.37
PT 01	12	4	3	2	0	1	0	22	3.26
ESP 01	28	4	5	3	0	2	0	42	6.22
ESP 02	20	4	3	2	0	1	0	30	4.44
TB 01	17	3	2	4	0	1	2	29	4.30
TB 02	17	5	3	2	1	2	1	31	4.59
MB	23	3	2	0	0	1	0	27	4.00
MR 01	18	8	5	4	1	4	3	43	6.37
MR 02	23	2	4	3	0	0	0	32	4.74
PBC 01	17	1	1	2	1	0	0	22	3.26
PBC 02	20	2	1	4	0	0	2	29	4.30
Lu 01	22	3	2	4	1	0	0	32	4.74
FS 01	16	4	4	2	0	0	2	28	4.15
KB	20	3	5	4	0	2	4	38	5.63
Total	427	53	69	72	7	20	29	675	100.00
In %	63.26	7.85	10.22	10.67	1.04	2.96	4.30	100.00	

### Characteristics of microplastics

As per the identification factor, MPs have a dimensional range of more than 1 μm and less than 5 mm, produced from the larger plastics through several degradations (Lee et al. 2021; Rodríguez-Narvaez et al. 2021). By looking into these size ranges, the extracted MPs were classified into five types having the scale ranges of 1–1000 μm, 1000–2000 μm, 2000–3000 μm, 3000–4000 μm, and 4000–5000 μm. For the monsoon samples, the distribution of MPs according to their size range for respective sampling sites is shown (Supplementary Fig. 4), both in number and percentage. From the mode of the size of distribution of MPs, it is found that particles having a size range 1000–2000 μm occurred as the most dominant, with a concentration of 26.77% in the region. Similarly, during the post-monsoon season, the distribution of MPs was observed. Particles having a size range 1000–2000 μm occurred as the most dominant, with a concentration of 24.45% (Supplementary Fig. 5). In addition, with the seasonal variation of the MPs occurrences and distribution during pre-COVID, post-COVID samples were analysed, to correlate the distribution of MPs according to their sizes. Notably, particles with a size range 1–1000 μm were the most dominant, with a concentration of 53.48% in the study area (Supplementary Fig. 6).

**Fig. 2:**
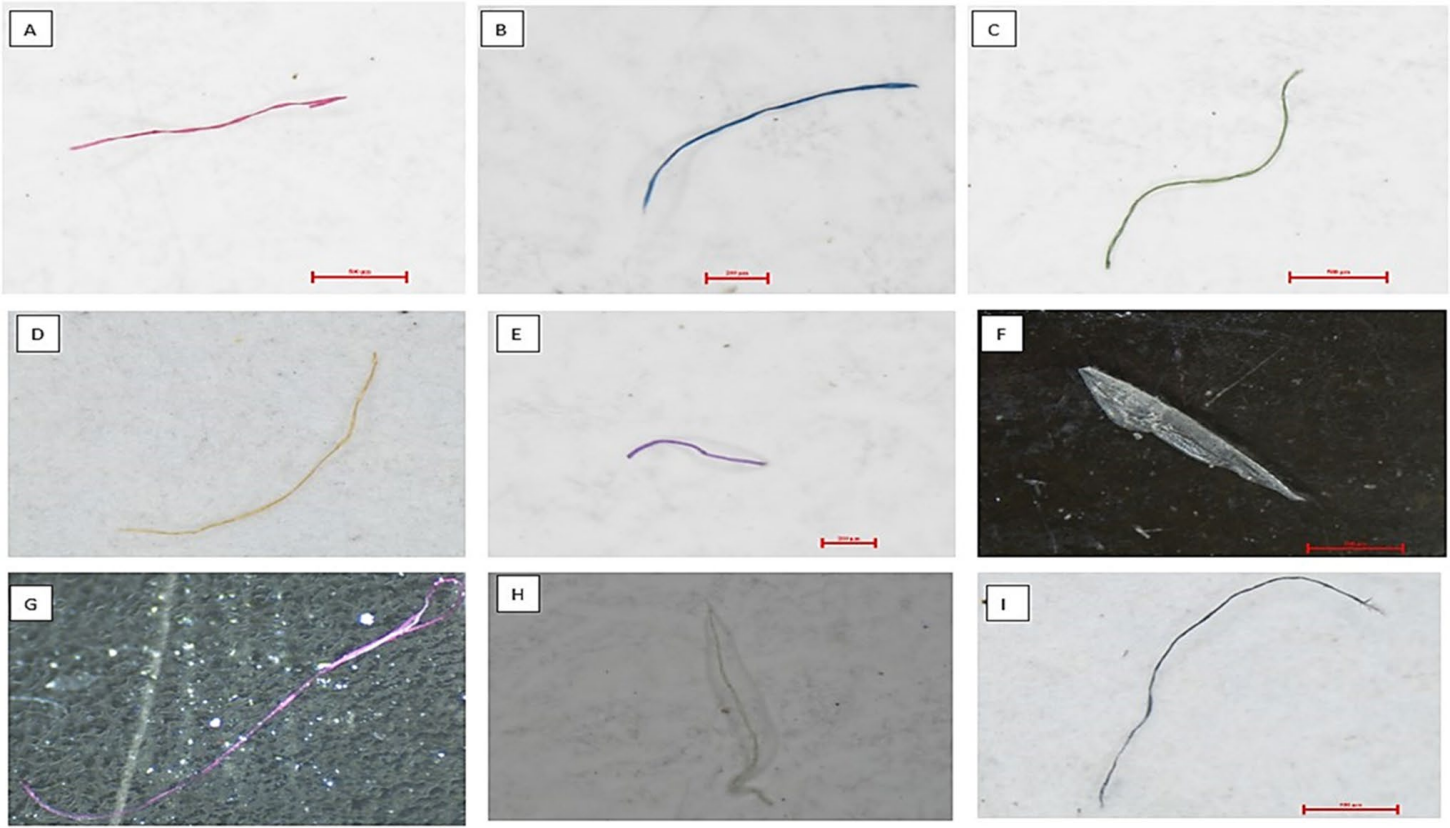
Image of several colours of MPs determined from beach sediments—(**A)** red, (**B)** blue, (**C)** green, (**D)** yellow, (**E)** violet, (**F)** transparent, (**G)** pink, (**H)** white, (**I)** black

A wide variety of colours in MPs are detected in the study area, which varies from place to place and season-wise. A total of 11 colours of MPs are observed; out of them, eight colours are more dominant, such as red, blue, green, yellow, violet, white, black, transparent, and others (including pink, brown, and grey). Photographs captured of several colours of MPs by Nikon-SMZ745 stereomicroscope are illustrated (Fig. 2). During the monsoon, the most dominant colours of MPs are blue (29.35%), followed by red (23.23%), black (14.84%), white (12.26%), yellow (7.74%), green (7.10%), transparent (3.23%), violet (1.29%), and others (0.97%) (Supplementary Fig. 7). Likewise, blue colour is the most dominant colour occurred with 29.26%, which is followed by red (27.51%), black (12.23%), white (11.79%), yellow (9.39%), green (4.80%), transparent (2.62%), and others (2.40%), detected during post-monsoon (Supplementary Fig. 8). It is noticed that in both seasons blue colour MPs are the most dominant colour, followed by red and black. Transparent or colourless MPs are the most dominant variety of MPs detected from the post-COVID samples, with a concentration of 29.33%, which is followed by blue (17.78%), white (17.19%), others (9.04%), black (7.26%), red (6.52%), yellow (6.52%), violet (4.59%), and green (1.78%) (Supplementary Fig. 9).

**Fig. 3:**
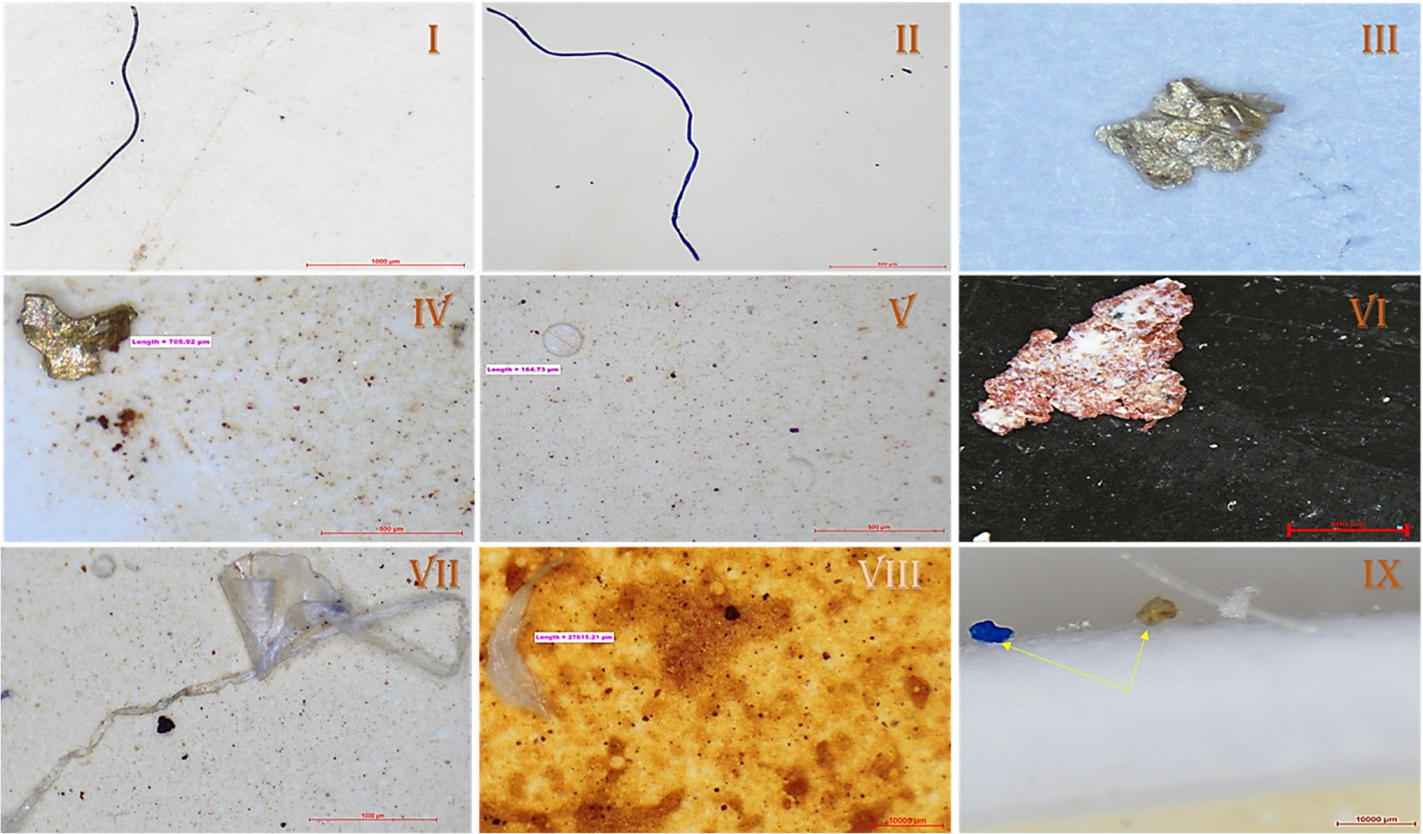
Images of different shapes of MPs—(I and II) fibres, (III and IV) fragments, (V) pellet, (VI) sheet, (VII) film, (VIII) foam, (IX) microbeads

The distribution of MPs based on shapes is an essential characteristic of MPs, where six common types are detected, such as fibre, fragment, pellet, sheet, foam, and film (Kelly et al. 2021; Ramkumar et al. 2022), along with these some microbeads were also encountered. In this current study, all the types of MPs were also identified during the analysis. The images of several shapes of MPs detected during stereomicroscopic observation are illustrated (Fig. 3). Out of the whole findings of monsoon samples, fibre shape MPs accounted as the most dominant type with a concentration of 68.39%, followed by beads (13.87%), fragment (9.03%), film (3.87%), foam (3.23%), and sheet (1.61%) (Table 1). Fibre shape occurred as the highest number of MPs type with a concentration of 70.74%, which is followed by beads (13.32%), fragment (7.86%), film (3.28%), sheet (2.84%), and foam (1.97%). The shapes of MPs during the post-monsoon season are determined from each sampling site of the 11 beaches of Miri (Table 2).

On the other hand, after the COVID-19 pandemic, similar analyses were carried out from these coastal sediment samples. From the ten remarkable famous beaches, a total of 23 sampling sites were chosen, out of which a sum of 675 MPs was extracted. Out of these 675 MPs, fibre MPs accounted as the dominant shape of MPs with a concentration of 63.26%, followed by foam (10.67%), fragment (10.22%), film (7.85%), beads (4.30%), sheet (2.96%), and pellet (1.04%) occurred as the least abundance MPs from the region (Table 3).

### Polymer identification of extracted MPs

**Fig. 4:**
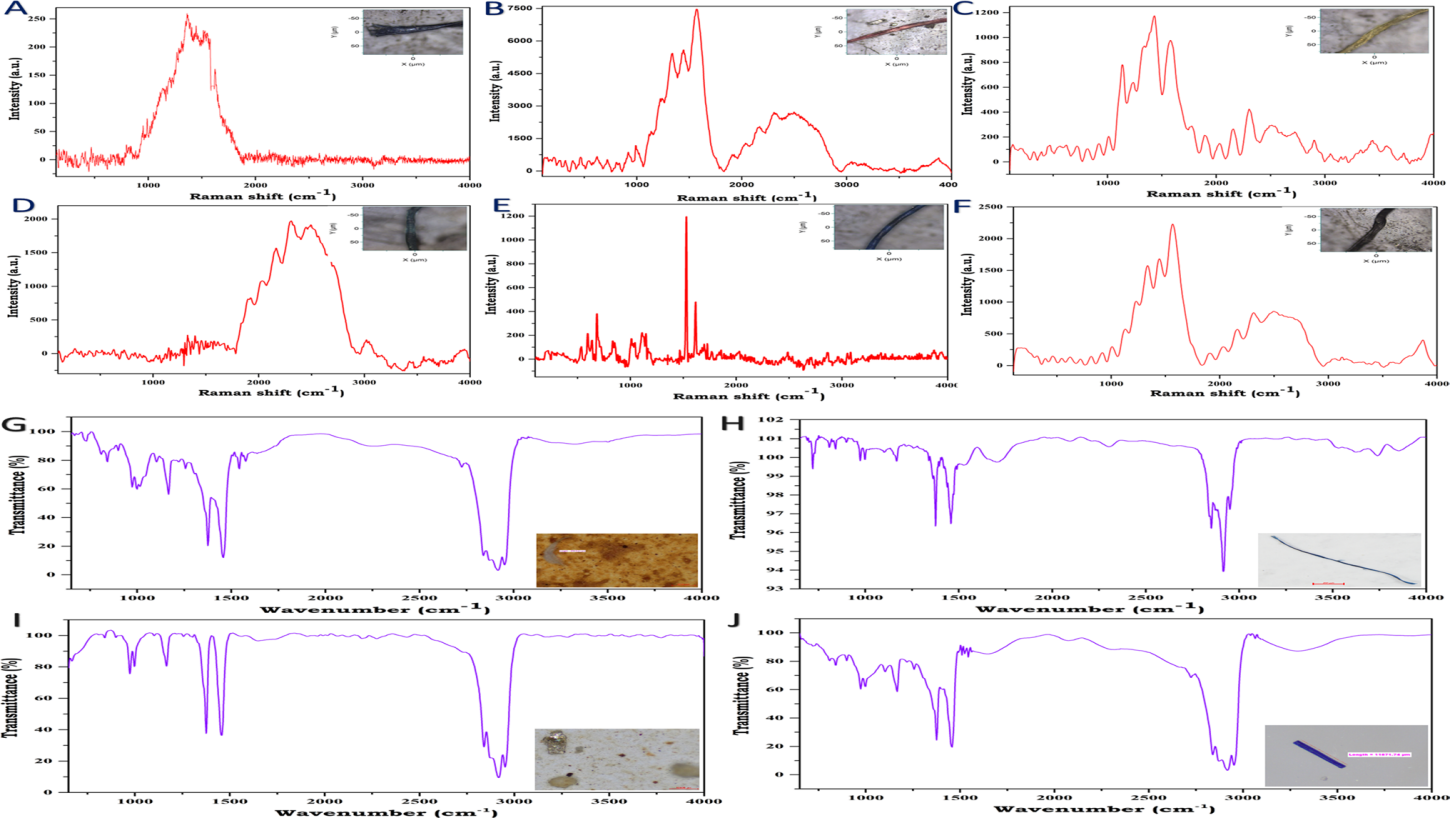
Pre-COVID: Raman spectra of the MPs showing the types of polymers attached with their respective image of MP: **A** PTFE; **B** PTFE and PET; **C** PET and PVC; **D** ABS; **E** PET; **F** PTFE and nylon. Post-COVID: ATR-FTIR spectra of the MPs showing the types of polymers attached with their respective image of MP: **G** nitrile; **H** PP and PE; **I** and **J** PE

After completing the stereomicroscopic analysis of the extracted MPs, the polymer composition of the MPs study was also carried out using Raman spectroscopy analysis for pre-COVID samples and ATR-FTIR analysis for the post-COVID samples. The selection of MPs was made based on their size, types, and colours from each sampling season. For the polymer composition, MPs were chosen from each colour and size range (one MP from each size category) from the pre-COVID samples. However, in terms of type, only fibres, fragments, and films were taken for analysis as the removal of MPs like beads, foams, and sheets from the filter papers was not possible. Another plausible reason for not considering the bead sized MPs were due to their less abundance. The polymer composition of a few selected MPs extracted from these two analyses is represented (Fig. 4), i.e. from both pre- and post-COVID periods. It was found that most of the polymers were composed of polytetrafluoroethylene (PTFE), polyester or polyethylene terephthalate (PET), polyethylene (PE), polyvinyl chloride (PVC), nylon or polyamide (PA), polystyrene (PS), and polypropylene (PP). It was identified that several MPs showed similar sizes and colours; however, their polymer types were different by showing different spectral bands. One spectral result demonstrated a peak at 1635 cm^−1^ (C=C bond stretching) and 1000 cm^−1^ (C–H bond), similar to a PS. Similarly, another results showed a peak at 2940 cm^−1^ (strong bands of CH_3_ asymmetric vibration), 1455 cm^−1^ (asymmetric bending of CH_3_), and 1350 cm^−1^ (C–H bending), representing PP (Jaafar et al. 2021). Likewise, the most distinctive Raman peaks for ABS occurred within 2800 to 3050 cm^−1^ (represents C–H stretching), and another prominent peak was generated within 2200 to 2300 cm^−1^ (C–N stretching) (Lenz et al. 2015). Another Raman spectra of MPs show peaks at 1730 cm^−1^ (ester C–O stretching), 1450 cm^−1^ (symmetric deformation of CH_2_), and within 600 to 700 cm^−1^ (stretching vibration of C–Cl) (Gillibert et al. 2019; Ripken et al. 2021). The polymer compositions also revealed that PTFE type occurred as the highest abundance, with a concentration of nearly 36%, followed by PE (23.45%) and PA (18.76%).

The post-COVID samples were analysed to identify the polymer types of MPs using attenuated total reflectance-Fourier transform infrared spectroscopy (ATR-FTIR). The ATR-FTIR spectra of the MPs are demonstrated (Fig. 4), through which the polymer compositions were determined. MPs were selected based on their size ranges, types, and colours for the spectroscopy analysis. One of the MPs represented peaks at 2928 cm^−1^ (asymmetric stretching of CH_2_), 2859 cm^−1^ (symmetric stretching at CH_2_), 1481 cm^−1^ (bending deformation), and 710 cm^−1^ (rocking deformation) was determined as PE (Choong et al., 2021). Similarly, another polymer was detected as PET, which represented the peaks at 3067 cm^−1^ (C–H symmetric stretch absorption bands), 1741 cm^−1^ (C=O stretching of the carboxylic acid group), 1471 cm^−1^, and 1342 cm^−1^ (C–O group stretching and ethylene glycol segment) (Choong et al., 2021). It was noticed that the most common polymer types were polyethylene (PE), polypropylene (PP), polystyrene (PS), polyethylene terephthalate (PET), high-density polyethylene (HDPE), nitrile, and cellulose or cellophane. Among these polymer types of MPs, the most dominant polymer was PE, followed by PP, PS, HDPE, PET, etc. During the post-COVID sampling, several PPE kits were collected and analysed the types of polymers; this is because of the predominance of this medical waste in the study area. Due to the COVID-19 pandemic and the guidelines of WHO, human beings adopted plastic-based PPE kits, especially disposable face masks, gloves widely and because of its mismanagement in the waste disposal treatment, they pollute the surrounding ecosystems (De-la-Torre 2020).

### Morphometric analysis of MPs

The scanning electron microscope (SEM) was used to determine the morphologies of the polymers through which the possible point sources of the MPs were determined (Ramkumar et al. 2022), as well as the elemental concentration of the selected MPs was also found through the peaks of energy dispersive X-ray spectroscopy (EDX) results (anak Alexander Tampang and Mohan Viswanathan 2022). For this analysis, a few MPs types were chosen, like fibres, fragments, foams, and films, whereas other types were excluded due to the less abundance and difficulty to remove from the filter papers, just like polymer identification tests. The microphotographs or SEM images of MPs are demarcated by showing their morphologies (Supplementary Fig. 10). It was observed that the morphology of fibre MPs was linear, smooth, and rounded (in broad view), whereas, for the film, it was an uneven and flat surface. For fragment MPs, the surface was uneven and rough; conversely, the surface was rounded and uneven for foam MPs. The microphotographs of MPs showing their morphologies and the EDX spectra of the respective circular marks are illustrated (Supplementary Fig. 11), showing the concentration of elements present within that specific type of MPs. From the EDX spectra, it was revealed that for the fibres and fragments, the average weight percentage of C varies between 35 and 50%, followed by O (35–45%), N (10–25%), and Ag (10–15%), whereas for the fragment, foam, and film type MPs the average weight percentage of C (32–40%), followed by O (25–32%), N (15–23%), Ag (8–13%), and Mo (1–4%).

## Discussion

### Occurrence and characterisation of microplastics

The occurrence of MPs in the marine environment is an emerging issue in this modern age, where these pollutants have been escalating the pollution level of this ecosystem day by day (Lin et al., 2022). The occurrence of MPs in the study area based on their size ranges varies widely, but it has been noticed that during pre-COVID (including monsoon and post-monsoon), the predominant MPs were within the ranges of 1–2 mm, followed by <1 mm, whereas during the post-COVID sampling, there was a big jump in the concentration of MPs of size ranges <1 mm (having 53.48% dominance), which is similar with other studies’ findings of the dominance of MPs sizes (Yaranal et al. 2021; anak Alexander Tampang and Mohan Viswanathan 2022). The higher abundance of MPs within these small ranges in the study area has several reasons for the degradation of larger plastics through various ways, such as exposure to UV and/or industrial needs to make commercial products (Vetrimurugan et al. 2020). These smaller MPs impact the habitats of all types of ecosystems, as they can transport a long way by wind and water current due to their lightweight (Liong et al. 2021). The occurrence of several colours in the MPs can be helpful in identifying the level of weathering or transportation to the depositional environment (Vetrimurugan et al. 2020), which clearly indicates that the higher the migration of MPs, the lesser will be the intensity of colour or they become fade to transparent type. In the current study, the most dominant colours shown by the MPs are blue, red, transparent, and black. Generally, packaging products like bags, fishing nets, and plastic bottles are manufactured with the help of transparent MPs (Yu et al., 2018; Zhu et al. 2021). For the manufacture of personal care products, coloured packaging, and washing ropes, several colours like black, blue, green, yellow, red, white, pink, brown, and purple colours are used with the MPs (Liong et al. 2021).

In the current study, all seven shapes of MPs were extracted from the Miri coastal sediments, out of which fibre-type MPs occurred as the most dominant type of MPs throughout the study. It was found that the concentration of fibre MPs occurred as 68.39%, 70.74%, and 63.26% from the monsoon, post-monsoon as well as post-COVID samples, respectively. It has been observed that the manufacturing of ropes, fishing lines, gears used for fishing, synthetic clothing, and other fabric products are the main reasons for the formation of fibre-type MPs (Sang et al. 2021; Choong et al. 2021b). Fragment-type MPs originated due to the degradation and deterioration of larger plastic products (Sang et al. 2021). The concentration of fragment-type MPs was detected as 85.1% and 46% from the recent studies on the Brazilian coast and India (Maynard et al. 2021; Yaranal et al. 2021). Pellet and microbead-type MPs were usually generated from personal care products like scrubs and facial cleansers, as demonstrated in recent studies (Liong et al. 2021; Fiore et al. 2022). A study also illustrated that food packaging, plastic bags, ornaments of clothing, and other synthetic fabrics products are the major reasons for the formation of film and pellet-type MPs (Sang et al. 2021).

In the present study, it is found that the occurrence of MPs in the coastal sediments varies according to several factors like transportation and degradation of larger plastics to micro level through fluvial or drainage disposal action, or by the UV mechanism, or by the seasonal variation, or due to the illegal waste disposal treatments by industries and local people in the marine environment (Noik and Tuah 2015). The current study finds that either near the river mouth or near the industrial port or any recreational activities beaches regions, the concentration of MPs is much higher than on regular beaches. Apart from that, there may be a possibility of discharge of land-based pollutants through run-off of the two major rivers, Miri and Baram, which play an important role in increasing the abundance of MPs on the Miri coast. A major concern is rising for human health issues, those intaking the aquatic organisms from Miri regions for a long term due to the exposure of MPs in the food chain (Jaafar et al. 2021). Although the significant impact of MPs on human health remains uncertain, there are no strict regulations imposed by the government to control the MPs’ pollution in ecosystems and seafood (Smith et al. 2018; Jaafar et al. 2021).

### Spatiotemporal distribution of microplastics

In this study, the spatial distribution of the MPs is shown by collecting samples from several crowded localities of the Miri coastline from every sampling period. No appreciable changes were found in the land use and land cover of the region during the study period (illustrated details in supplementary materials, i.e. Supplementary Table 1 and Figs. 1 and 2). Microplastic abundance was evaluated in pre-COVID samples, emphasising regional seasonal changes and post-COVID samples were compared to that baseline. Likewise, two common seasons are chosen to demonstrate the temporal distribution of MPs in the regions, i.e. monsoon and post-monsoon. Apart from that, by looking into the current scenario of global conditions due to the COVID-19 pandemic, ambitious work has been added by doing the whole process (sampling and all analyses of MPs) for the post-COVID samples, to demonstrate the pre-COVID and post-COVID variation of the distribution of MPs in the current study area. Even though the abundance of MPs varies seasonally, as per the current study, the fact is that these MPs are transported through major rivers like Baram, Miri, and Sibuti, forming the hotspots near their estuaries or nearby shorelines. It has been found that during monsoon, the hotspot or highest concentration of MPs extracted from Kuala Baram Estuary (KB O1—6.77%), similarly during the post-monsoon, the most abundant of MPs detected at Fish Landing Senadin (FS(II) 02—7.21%). Again, near Kuala Baram Estuary, a similar pattern was shown during the post-COVID sampling, where Miri River Estuary (MR 01—6.37%). The abundance of MPs increases in the marine environments due to the discharge of rivers into them or their small distributaries (Zhu et al. 2021). It has also been demonstrated in the results that the concentration of MPs gradually decreased from estuaries outwards, which means the land-based pollution discharge into the seas or oceans is also controlled by rivers and some drainages. As the tidal activity of the South China Sea near the Miri coast is quite fluctuating, the sampling was carried out according to the Miri meteorological data during the low tides (Rashidi et al. 2021). The outcomes of the study show that the average of MPs abundance during monsoon was 11 ± 3 particles per 30 g, and during post-monsoon, it was 15 ± 5 particles per 30 g, explaining about pre-COVID finding. The pollution level or concentration did a big jump in the post-COVID findings, which was 29 ± 5 particles per gram. These pre-COVID findings demonstrated an inverse relationship between the MPs’ pollution and the rainfall or monsoon seasons. The level of MPs concentration increased by 148% during post-monsoon time than monsoon, and this amount was enormously increased during post-COVID. The occurrences of polymer types were also changed from pre-COVID to post-COVID, i.e. during the pre-COVID time, the most dominant polymers were PTFE, PET, and PE, whereas, after the COVID-19 pandemic, the predominance of polymer types were PE, PP, PS, and PET.

### Potential source materials determination of microplastics

The dominant polymer type PTFE occurred from the pre-COVID results of the current study, which is widely used in the oil and gas industries, non-stick coating, chemical processing industries, and electrical industries. The second highest dominance of polymer type in the current study was PE, usually used in food and beverage packaging, packaging bottles such as soft drinks bottles, water bottles, and shampoo bottles (Choong et al. 2021a; Liong et al. 2021). Another dominant variety of polymers found throughout all the spatiotemporal studies is PET or polyester, commonly used in garment industries, woollen goods, and textile industries (Wang et al. 2017). Likewise, PS was the dominant variety of polymer found from the post-COVID sampling, generally used for boxes and food packaging (Liong et al. 2021). The two other varieties of polymers that occurred in the current study were PA and PP, whose potential sources are fisheries and aquaculture (Hamzah et al. 2021). It is also crucial to highlight that PVC is frequently combined alongside certain plasticisers, typically within the phthalates group, and used as a food-grade PVC film (Gillibert et al. 2019). The SEM-EDX results are the elemental composition attached to the MPs surface, which is useful to demarcate the possible manufacturing industries or point sources of the MPs. The additives used in the plastics for improving electrical resistance or radiation or durability, as well as the chemicals absorbed from the existing environments, are the dominant chemicals present in MPs (Campanale et al. 2020). Apart from that, to enhance the properties of the plastics during the manufacture of several products, stabilisers, fillers, and plasticisers are commonly used, which make these plastic products more durable to UV radiation and degradation processes as well as more toxic (Campanale et al. 2020). The most dominant elements detected in EDX analysis of the MPs extracted from Miri coastal sediments are oxygen and carbon, explaining they are constantly exposed to UV radiation, weathering actions as well as the increase of hydroxyl groups (Vetrimurugan et al. 2020; anak Alexander Tampang and Mohan Viswanathan 2022). The occurrence of Al in a few of the MPs illustrates as the by-product of inorganic pigments or stabilisers or flame retardants, and this is usually found in PE, PET, or PVS polymers; similarly, the Cl element found in PVC (Wang et al. 2017). The occurrence of silver (Ag) in the MPs indicates the product of pharmaceutical industries or jewellery or dressing industries; similarly, titanium (Ti) in a few of the MPs are usually used as TiO_2_ indicator white pigment or UV stabilisers (Campanale et al. 2020; Vetrimurugan et al. 2020). Furthermore, in most cases, the metals or elements detected through EDX analysis and the polymer types and shapes are the indicators of determination of possible sources of MPs. However, the additives or elements are used to make the plastics more durable to degradation and resistant to UV radiation.

### The role of COVID-19 on microplastics pollution

In the current century, plastic pollution is emerging as a transboundary threat to human civilisation as well as to the natural ecosystems concerning its disposal, whether it is in the form of macro or micro or nano plastic forms. During the COVID-19 pandemic, it has been noticed that the use of personal protective equipment (PPE kit including a face mask, gloves, and gown) has been enormously increased due to the guidelines of WHO (World Health Organisation) as well as to protect ourselves from the deadly SARS-CoV-2 viruses; although these are necessary steps for the time beings, these PPE kits are manufactured from single used plastics (Kwak and An 2021). Several polymers are used to manufacture these PPE kits (mainly used for making face masks), like polypropylene (PP), polyurethane (PU), polyacrylonitrile (PAN), polyester (PET), polycarbonate (PC), polystyrene (PS), or polyethylene (PE) depending on the brand and requirement of customers, whereas nitrile and/or latex are used to produce disposable gloves (Aragaw 2020; Ammendolia et al. 2021). The excessive use of single-use face masks and PPE kits leads to the enormous disposal of these wastes to the surrounding ecosystems, and due to the lack of research on their disposal process or reuse techniques, they became emerging pollutants in the ecosystems (Teymourian et al. 2021). Apart from this, during the pandemic, the excessive use of vaccine units and medical care products produced considerable medical waste, which increased the hazardous waste in the environment and the possibility of infection (Dharmaraj et al. 2021). An estimation revealed that in 2020, nearly 1.56 billion face masks were discharged into the oceans (Akber Abbasi et al., 2020).

**Fig. 5:**
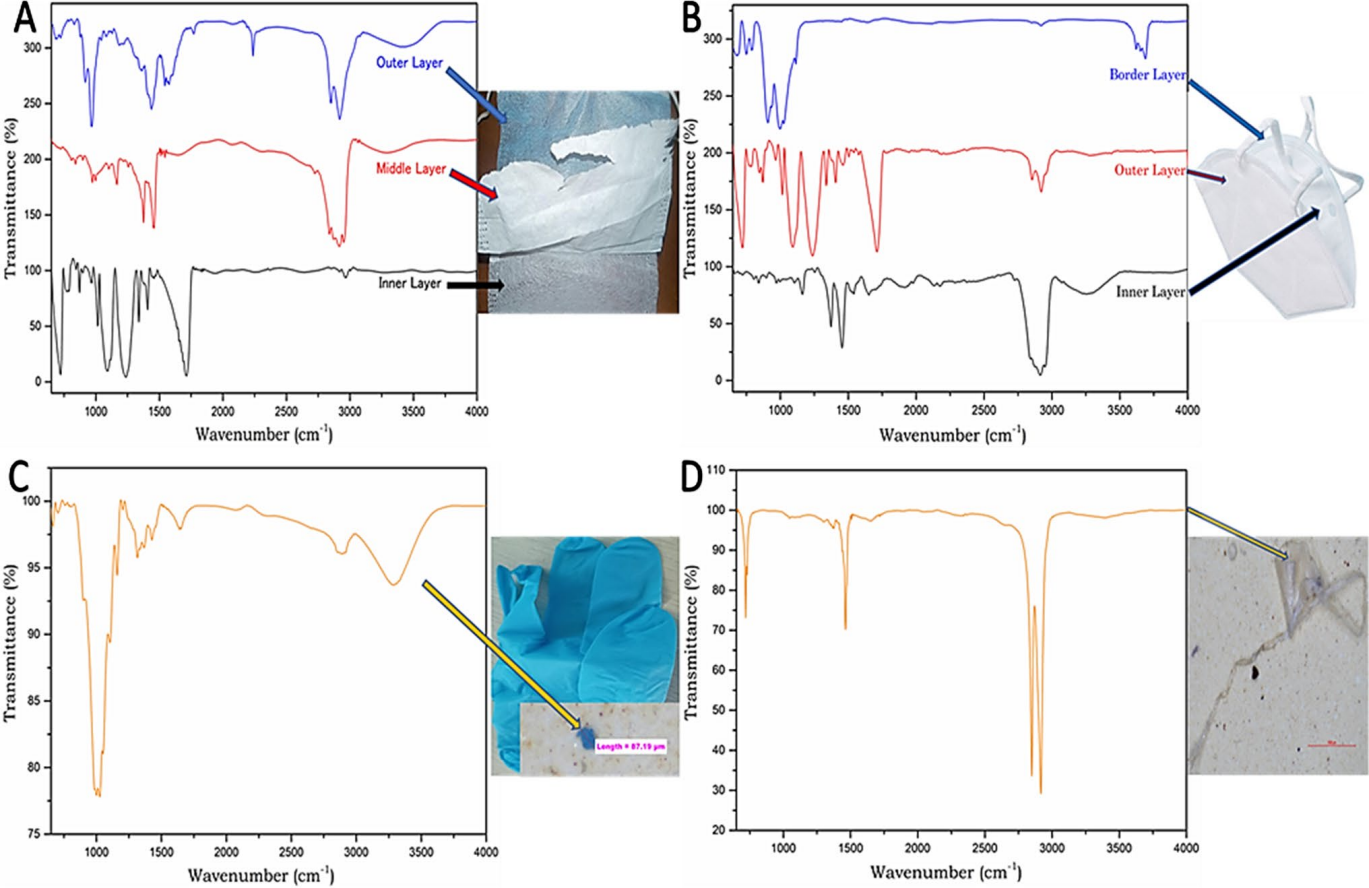
Comparative study of ATR-FTIR spectra of different layers of face masks: **A** PS (inner layer), PP (middle layer), PP and PS (outer layer); **B** PP (inner layer), PS (outer layer), cellophane (border); ATR-FTIR spectra of few PPE kits: **C** nitrile (gloves); **D** PE

In the current study, during the sampling activities for the post-COVID time, it has been noticed that the coastal regions were much more polluted with plastic products such as face masks, gloves, food and beverages packets, and tyres, and ultimately due to the degradation of the plastic waste, MPs are produced enormously. The results have revealed that the MPs extracted (*n* = 675) from the sediment samples of post-COVID from the Miri coast are 148% higher than the post-monsoon (*n* = 458) and similar 218% higher than the monsoon (*n* = 310), although, during post-COVID sampling, only 23 number of sampling sites are selected which is comparatively lesser than post-monsoon and monsoon sampling sites, i.e. 30 and 26, respectively. Figure 5 shows the comparative study of ATR-FTIR spectra of different layers of face masks: A—PS (inner layer), PP (middle layer), PP and PS (outer layer); B—PP (inner layer), PS (outer layer), cellophane (border); ATR-FTIR spectra of few PPE kits: C—nitrile (gloves); D—PE (Aragaw 2020). The face masks and gloves collected during the sampling were carried out through ATR-FTIR spectral analysis, and the peaks are exclaimed by matching with the earlier study. It has been noticed that N95 masks and surgical face masks can produce microfibres once they enter the ecosystem, confirmed by two recent studies (Aragaw, 2020; Fadare and Okoffo, 2020). Another recent study was conducted with five different face masks (including disposable and reusable varieties), which revealed that by washing these face masks in a domestic washing machine, they released an average of 284.94 ± 73.66 synthetic or natural fibres into the ecosystem (De Felice et al. 2022). Although the occurrences and abundances of face masks and other PPE kits in several ecosystems are pretty common after the pandemic, there are very few studies and awareness promoted to show the sources of MPs, as personal care products and other plastic products are still the primary sources of production of MPs. This study will prepare a comparative demonstration of the growth of MPs and its impact in the Miri coastal area due to the COVID-19 pandemic; apart from that, more discussion should be done on this topic as well as government should take some fruitful action on the disposal or recycle of this pandemic waste.

### Series of evaluation and distribution of microplastics

A recent publication explained the impact of the COVID-19 pandemic on the ecosystem in Malaysia, especially correlating the MCO and municipal solid waste (MSW). From January to December 2020, data from the Solid Waste Management and Public Cleaning Corporation (SWCorp) was collected. The amount of MSW was reduced by the early MCO deployment across 41 local authorities, which make up 87.23% of Peninsular Malaysia. Nevertheless, MSW production increased again during the conditional and recovery stages (Brohan et al. 2021). The COVID-19 outbreak was for 2 years (i.e. February 2020 to January 2022) in the study area, and due to the MCOs, the front liners had restrictions to accomplish their tasks, which led to the increase of municipal solid waste on the coast. Further, an increase in fishing activities were also observed in some parts of the study area during the post-COVID, which added more polymeric pollutants in the regions with a decrease in industrial and tourism activities. The most significant contributors to the increase in MPs’ pollution in the ecosystem were households, agricultural, and medical waste (Facciolà et al. 2021; Brohan et al. 2021; Bilal et al. 2023; Ruíz-Reyes et al. 2023). Apart from this, due to the non-availability of biomedical waste disposal data neither during nor after the pandemic in the regions is a limitation of the study. Based on the earlier studies (considered as pre-COVID), during COVID (anak Alexander Tampang & Mohan Viswanathan, 2022), and a recent study (considered as post-COVID) in the Miri coast, a comparative approach to the occurrence of MPs can be prepared. It has been noticed that during the earlier studies, the temporal distribution of MPs was demonstrated by analysing the samples of two different seasons. This earlier sampling is considered a pre-COVID study, where the occurrence and distribution of MPs show that the post-monsoon (*n* = 458) season is more contaminated by MPs than the monsoon (*n* = 310) season, whereas, during the COVID, the MPs’ (*n* = 1305) pollution level is the highest compared to the recent study (considered post-COVID, *n* = 675). Earlier and recent studies found that the most abundant places containing MPs are near the estuaries. However, during the COVID-19 pandemic, the highest abundance of MPs is detected in Lutong beach, one of the recreational activity zones on the Miri coast (anak Alexander Tampang and Mohan Viswanathan, 2022). The most dominant shape of MPs is fibre-type (with an avg. concentration of 66%) identified in both pre- and post-COVID analyses. In contrast, fragment-type MPs (concentration of 60%) are the most dominant shape during the pandemic. The most dominant polymer types during pre-COVID are PTFE, followed by PET and PE, although PE is the most dominant polymer type during and post-COVID scenarios. However, the elemental compositions are almost the same, with C and O occurring as the most dominant elements throughout the studies. A series of evaluations and distribution of MPs during these periods in the Miri coast show the growth of MPs in the region (Table 4).

**Table 4:** Comparative results of MPs showing the spatiotemporal distribution in the Miri coast

Sampling period		Number of samples	Method of extraction	Most abundant location	Abundance (particles/unit)	Dominant shapes	Types of polymers	Common elements
Earlier studies/pre-COVID	Monsoon (December 2013)	26	Density separation by ZnCl_2_	Kuala Baram	21 particles/30 g	Fibres and fragments	PTFE, PET, PE, PVC	C, O, N, Ag, Al
	Post-monsoon (June 2014)	30	Density separation by ZnCl_2_	Fish Landing Senadin	33 particles/30 g	Fibres and fragments	PTFE, PET, PE, PA	C, O, N, Ag, Mo, Al
During COVID (2021)		24	Density separation by ZnCl_2_	Lutong beach	327 particles/90 g	Fragments and fibres	PE, PET, PS, PP	C, O, Ca, Al, Ti, Cl
Recent study/post-COVID (July 2022)		23	Density separation by ZnCl_2_	Miri River Estuary	43 particles/30 g	Fibres and fragments	PE, PP, PS, PET, HDPE	C, O, Si, Al, Fe, Cl, Mg, Zn, Na

### Statistical analysis

**Fig. 6:**
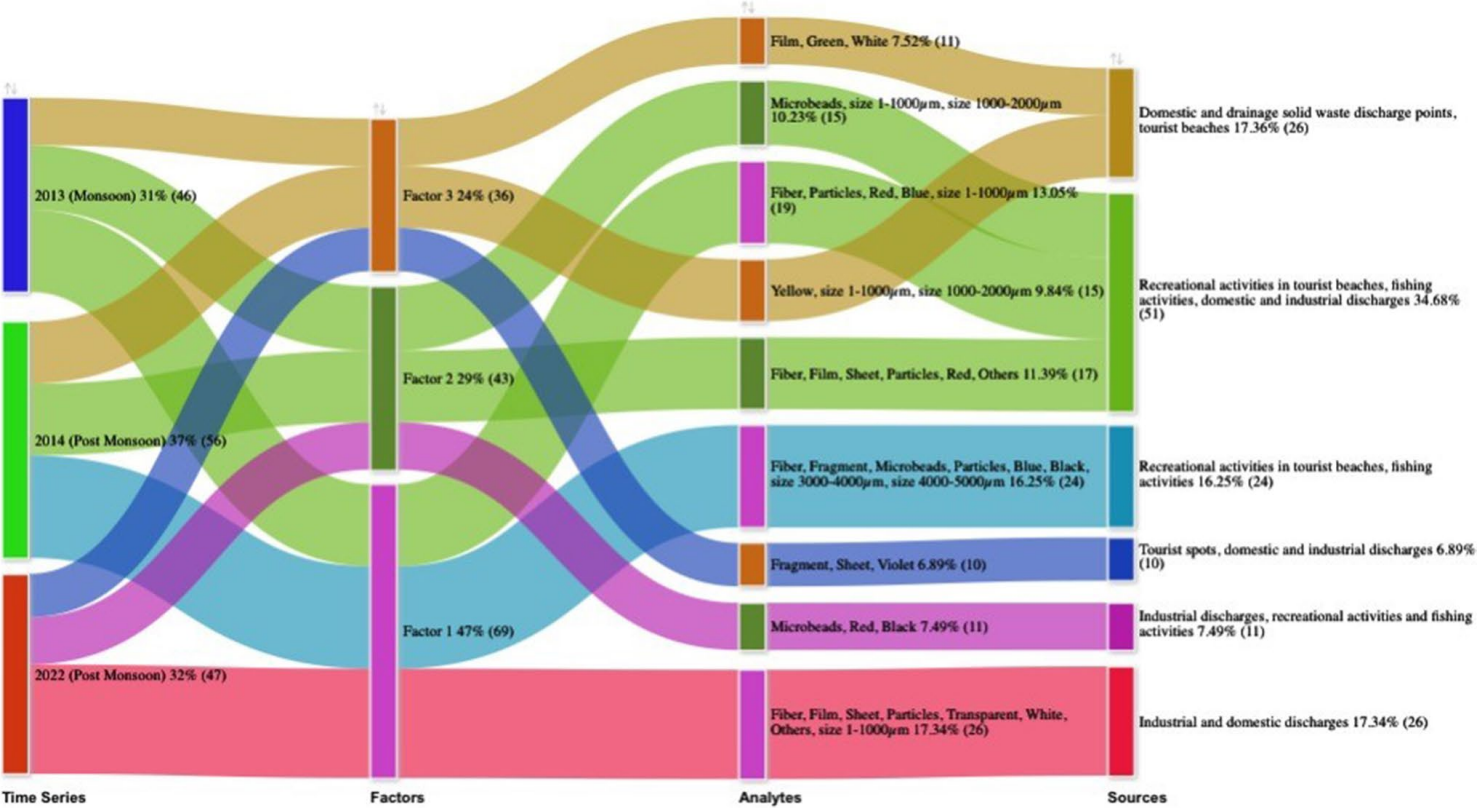
The summary of potential sources of MPs with respect to PCA analysis for the three different sampling periods

Principal component (PC) analysis was used to examine the prevalence and the relevant sources of MPs at different sample sites along the Miri coast. MPs characteristics such as colours, shapes, and sizes were used as variables for the three different sampling periods, and the factors were extracted with the factor scores. The varimax rotation was utilised and the components having eigenvalues >1 were considered for interpretation (Rakesh Roshan et al., 2023). The first three dominant factors which control the occurrence of MPs in the study area were selected for each sampling period. Overall the significant contributions were 47% for PC1, 29% for PC2, and 24% for PC3, irrespective of sampling periods (Fig. 6). PC1 was loaded with fibre, fragment, film, sheet, microbeads, particles, transparent, white, others, blue, black, red, blue, size (1–1000 μm), size (4000–5000 μm), and size (1–1000 μm) from the three sampling periods. The possible sources of MPs were industrial and domestic discharges, recreational activities in tourist beaches, and fishing activities. PC2 was loaded with microbeads, fibre, film, sheet, particles, red, black, others, and size (1000–2000 μm) from the three sampling periods. The possible sources of MPs were recreational and fishing activities and domestic and industrial discharges. PC3 was loaded with fragment, sheet, film, green, white, yellow, violet, size (1–1000 μm), and size (1000–2000 μm) from the three sampling periods. The possible sources of MPs were domestic and drainage solid waste discharge points and tourist beaches. It is interesting to observe that all the possible sources of MPs through various activities occurred in all the three sampling periods. Nevertheless, the analytes of the post-COVID samples were characteristic of fibre, film, sheet, particles, transparent, white, others, and size 1–1000 μm in factor 1; microbeads, red, and black in factor 2; and factor 3 with fragment, sheet, and violet. This indicates the fact that though the contributing sources remain the same, the nature of MPs after COVID has changed.

### Possible ecological risk assessment of microplastics

Marine ecosystems are the ultimate sink for waste, with fibre MPs being significant pollutants. These pollutants increase their abundance, causing challenges for trophic levels. Fatal diseases, abnormal childbirth, oxidant imbalances, increased mortality, and physical deformities are observed (Cole et al. 2020; Zainuddin et al. 2022). Based on market requirements, MPs are manufactured from artificial polymers employing intricate processes and sometimes from hazardous chemicals. Polymers that are exposed to different conditions contaminate ecosystems via thermal decay, UV radiation, and adhesion to hazardous components. The UN’s Globally Harmonized System states that more than half of the compounds in polymers are hazardous (Lithner et al. 2011; Zainuddin et al. 2022). The potential ecological risk evaluation of MPs can be calculated by using the pollution load index (PLI), polymer hazard index (PHI), and potential ecological risk index (PERI) indicators. Four categories make up the risk level of MPs based on the estimated PLI values (hazard categories for respective parameters have been demonstrated in supplementary Table 2). The results of the PHI and PERI indices represent five categories of hazardous intensity for each environment (Li et al. 2020; Ranjani et al. 2021). The discovered polymers and their corresponding monomers, densities, hazard scores, or risk classifications are shown in Table 5 based on the complete categorisation of the toxicities and hazard scores by Lithner et al. (2011). Along with these, the respective polymers’ possible applications or potential sources have also been demonstrated (Lithner et al. 2011; Xu et al. 2018). PE and PP are the major polymer types of MPs identified in the study area, which has low hazard score. However, the abundance of these polymers could create the ecological risk.

**Table 5:** Information in-depth about the MPs (polymers) found in this study (data from Lithner et al. 2011; Europe: Plastics—The Facts 2016—Google Scholar; Xu et al. 2018)

Polymers	Monomers	Density (g/cm^3^)	Significant utilisation	Hazard score
Polyethylene (PE)	Ethylene	0.91–0.96	Straw/pipes, toys, houseware, shopper bags, bottles, etc.	11
Polystyrene (PS)	Styrene	1.05	Packaging, plastic containers, sun-glass frames, etc.	30
Polypropylene (PP)	Propylene	0.85–0.94	Microwave-proof containers, food packaging, etc.	1
Polyvinyl chloride (PVC)	Vinyl chloride	1.41	Cable covers, pipes, door/window profiling, etc.	10,551
Polyamide (PA/nylon)	Adipic acid	1.14–1.15	Pigment, automotive appliances, drug covers, etc.	47
Acrylonitrile-butadiene-styrene (ABS)	Styrene	1.02–1.08	Plastic pipes, toys, automotive appliances, etc.	6552
Polyurethane (PUR)	Propylene oxide	0.40–0.60	Packaging bags, automotive appliances, carpet, furniture, etc.	7384

## Conclusions

To conclude, the current study reveals the occurrence and widespread distribution of MPs at different periods in the Miri coastal region. The results obtained from the current study represent the spatiotemporal distribution of MPs in the Miri coastal environment along with the characterisation study of these toxic pollutants and a comparative study between pre- and post-COVID occurrence of MPs. The results show that the occurrence and distribution of MPs during the post-monsoon season is 148%, which is more than during the monsoon season. Similarly, during the post-COVID, the MPs distribution is the highest among all three periodical studies. The study also finds that the highest concentration of MPs occurs near the estuary regions or any industries where the discharge of waste, shipping activities, and recreational activities are more noticeable. The size distribution of MPs shows that the size ranges between 1 μm and 1 mm and 1 and 2 mm are more dominant in the study area due to the degradation and transportation of MPs from different ecosystems. The current study also explains the distribution of MPs based on their colours, which reveals that blue, red, transparent, white, and black colour MPs have dominantly occurred throughout the study. The result shows that fibres are the most dominant variety of MPs in all three time-frame studies, with more than an average of 64%. The other common types of MPs are identified as fragments, films, and microbeads. By analysing the extracted MPs, it is found that PTFE, PP, PE, PET, and PS are the most common polymer types in the Miri coast.

Furthermore, the morphological and elemental composition study by SEM and EDX analysis shows the potential sources of MPs in the study area. C, O, N, Al, and Ag are the most dominant elements demonstrated by the EDX results. The result shows that the surface of fibre MPs is linear, smooth, and rounded (in broad view), whereas, for the film, it is an uneven and flat surface. For fragment MPs, the surface is uneven and rough; however, for foam MPs, the surface is rounded and uneven. This morphometric analysis is carried out for selected MPs, which reveals the transportation and the possible origin of these pollutants in the region. From the PCA, it was inferred that industrial and domestic discharges, recreational activities in tourist beaches, and fishing activities are the dominant sources of MPs in the Miri beach sediments.

Hence, the local government should take a more systematic approach in the form of regulations proposed for waste disposal and treatment plants, risk assessment of MPs in the marine environment, and awareness among people about the adverse effects of these unnoticeable pollutants. This study also observed the influence of the COVID-19 pandemic on MPs occurrence. Overall, this study provides baseline data about the spatiotemporal distribution of MPs and their impact on the Miri coast as well as surrounding environments, which helps to determine sustainable management plans in the study area.

### Supplementary information

ESM 1

## Data Availability

The datasets used and analysed during the current study are available from the corresponding author upon reasonable request.
