# Supplementary material for: Spatiotemporal distribution of microplastics in Miri coastal area, NW Borneo: inference from a periodical observation
Source: Environ Sci Pollut Res Int. 2023 Sep 9;30(46):103225–43. doi: 10.1007/s11356-023-29582-7 (PMC10567912; doi:10.1007/s11356-023-29582-7)
Supplement: Supplementary file 1 — ESM 1 [file 11356_2023_29582_MOESM1_ESM.docx]

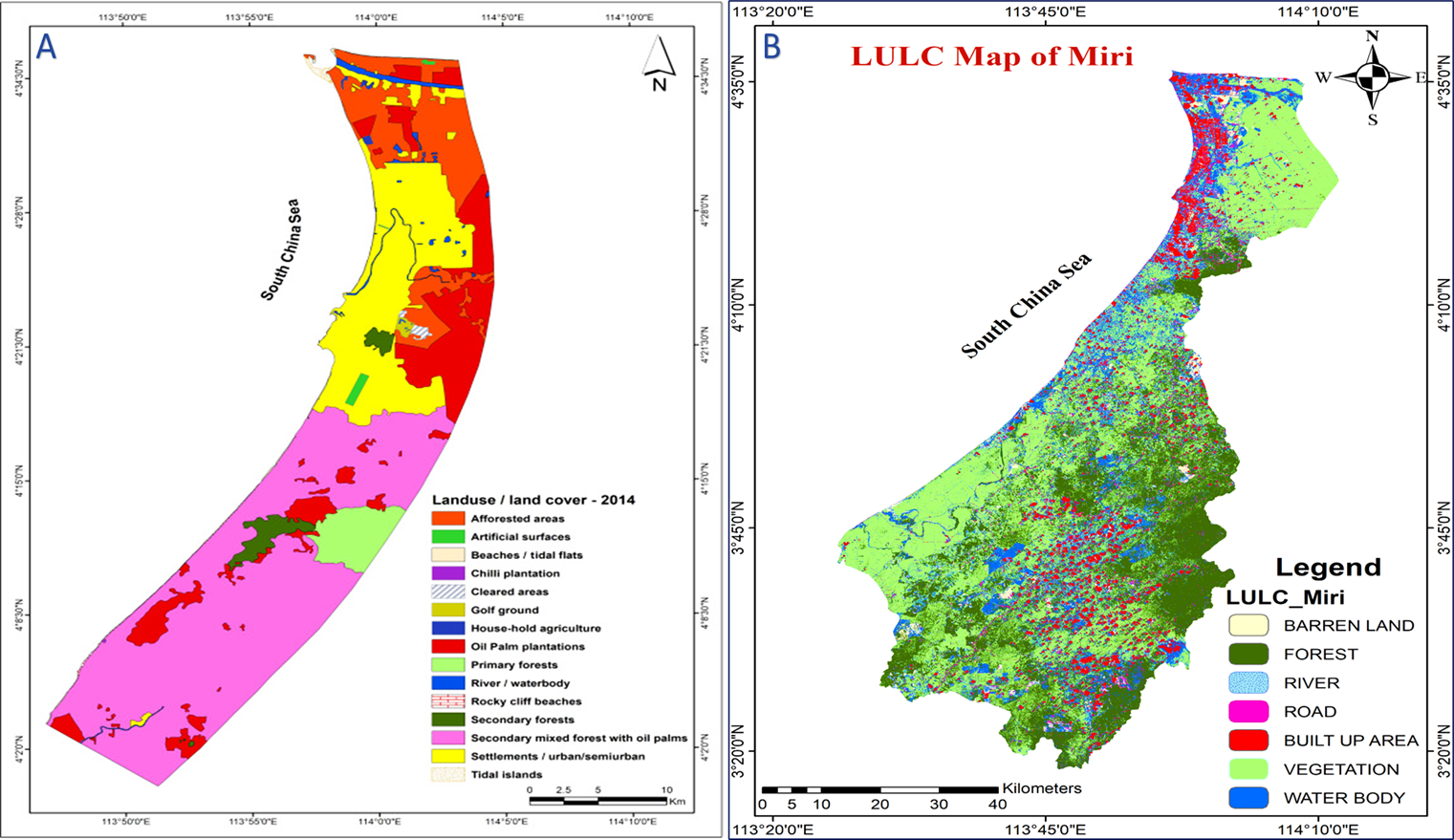


**Fig. 1** The LULC maps of the study area A represents 2014 (Anandkumar et al., 2018) & B represents 2022 (Landsat 8 data have been used in both cases)


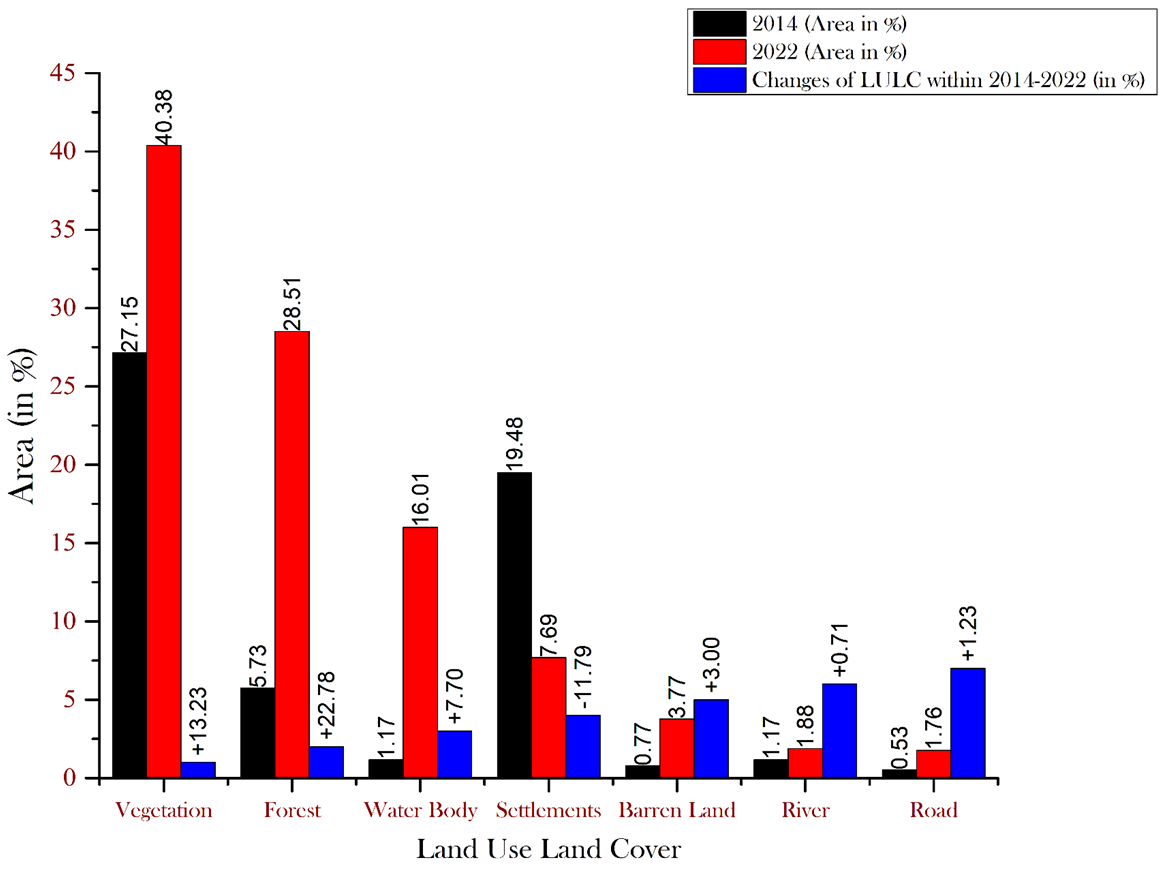


**Fig. 2** The LULC variation of the study area from 2014 to 2022

**Table 1** The variation of LULC types during 2014 (Anandkumar et al., 2018) and 2022

| **Land Use/Land Cover** | **2014** | | **2022** | | **Changes of LULC within 2014-2022 (in %)** |
| --- | --- | --- | --- | --- | --- |
|  | **Area (Km²)** | **Area (%)** | **Area (Km²)** | **Area (%)** |  |
| **Vegetation** | 521.05 | 27.15 | 1911.31 | 40.38 | +13.23 |
| **Forest** | 40.89 | 5.73 | 1349.47 | 28.51 | +22.78 |
| **Water Body** | 8.31 | 1.17 | 757.74 | 16.01 | +7.70 |
| **Settlements** | 138.97 | 19.48 | 363.75 | 7.69 | -11.79 |
| **Barren Land** | 5.46 | 0.77 | 178.45 | 3.77 | +3.00 |
| **River** | 8.31 | 1.17 | 88.78 | 1.88 | +0.71 |
| **Road** | 3.77 | 0.53 | 83.48 | 1.76 | +1.23 |


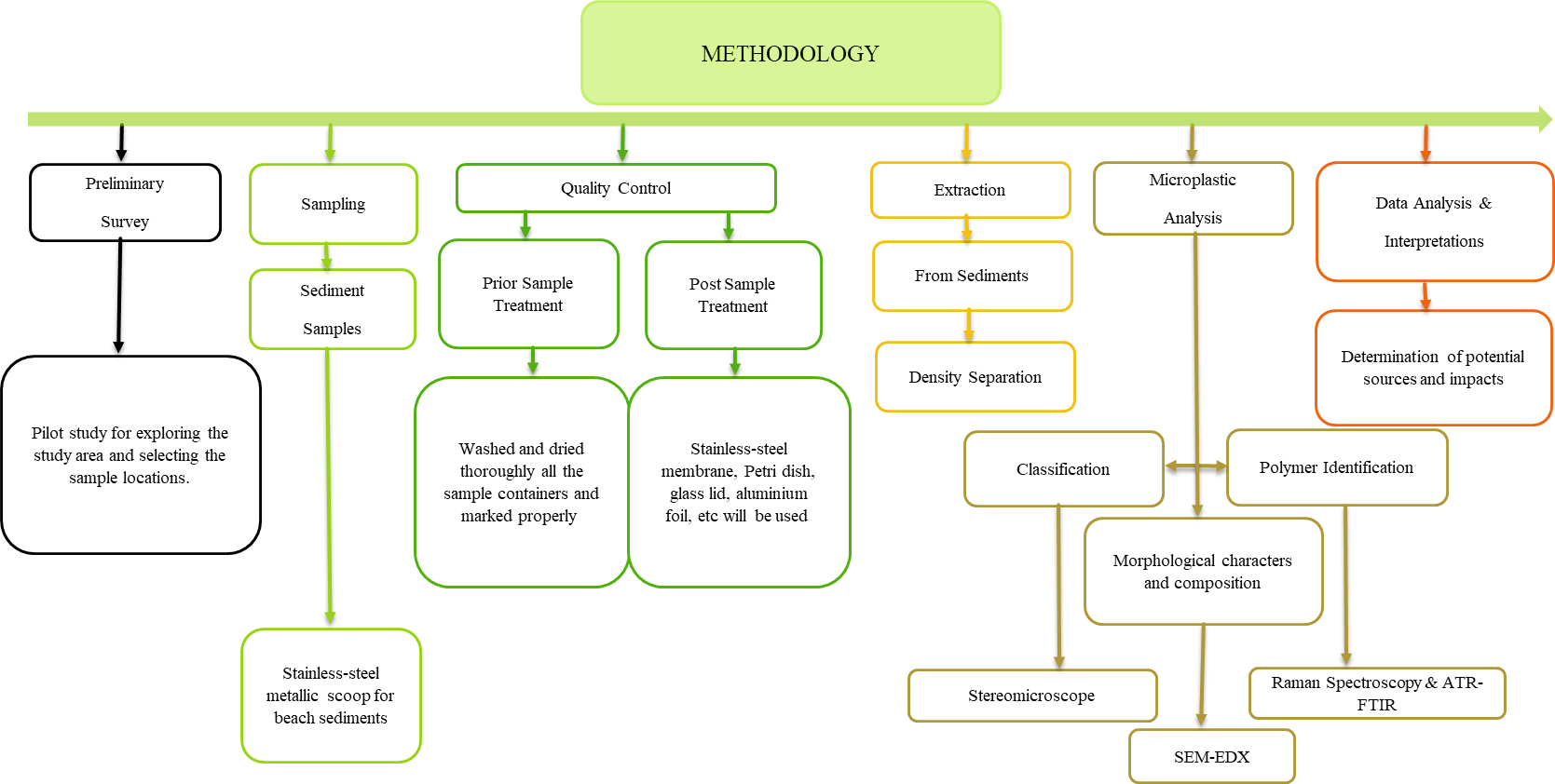


**Fig. 3** Flow chart showing the complete research methodology.

***
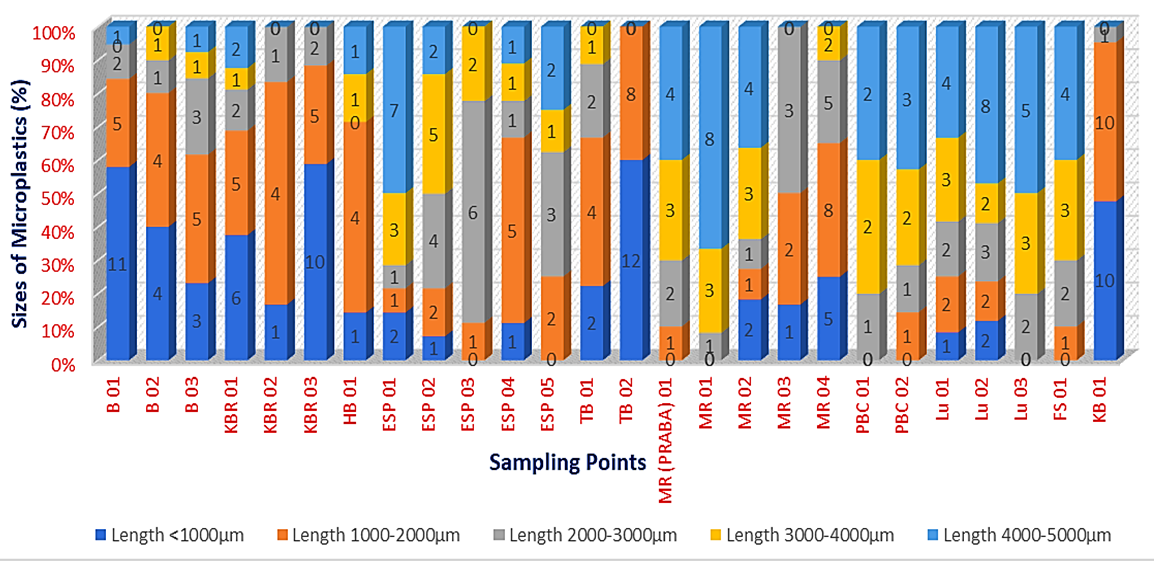
***

**Fig. 4** Graph shows the distribution of MPs according to their size variation (Monsoon)


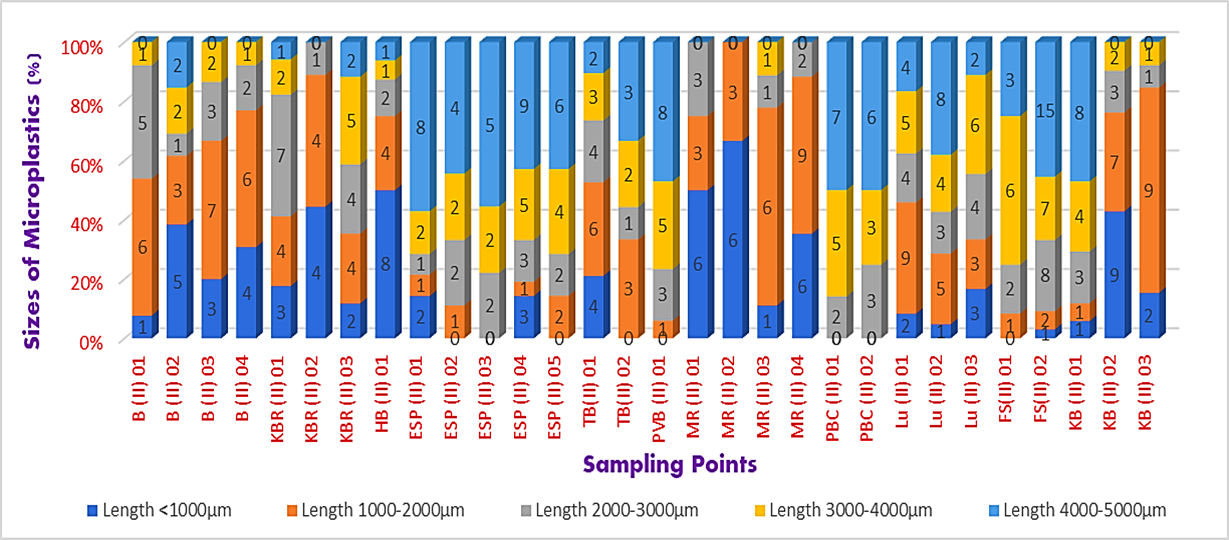


**Fig. 5** Graph shows the distribution of MPs according to their size variation (post-Monsoon)


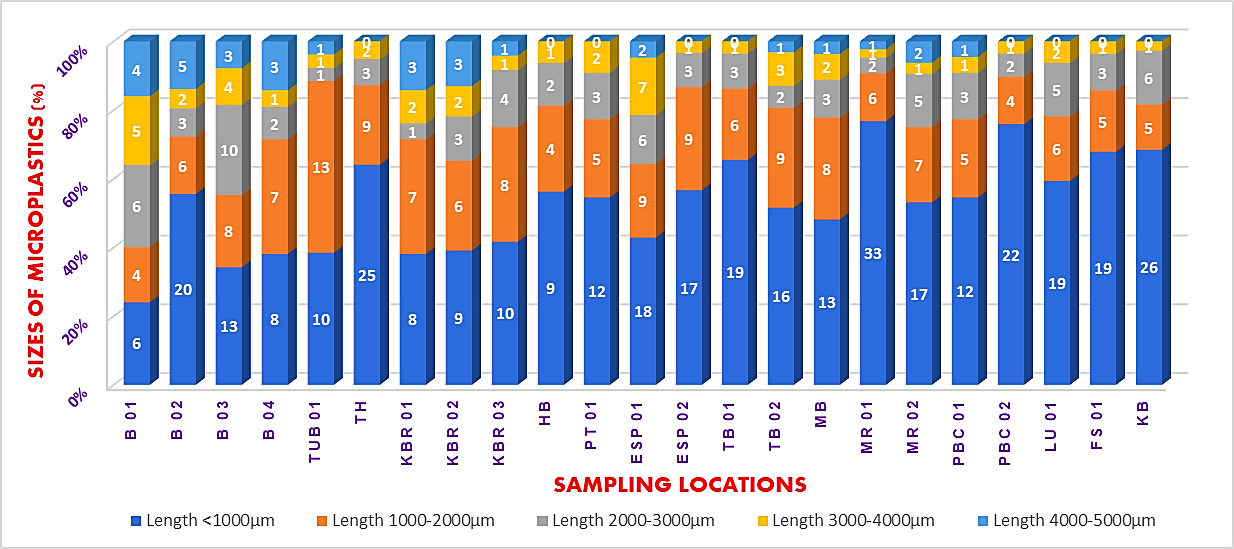


**Fig. 6** Graph shows the distribution of MPs according to their size variation (Post-COVID)


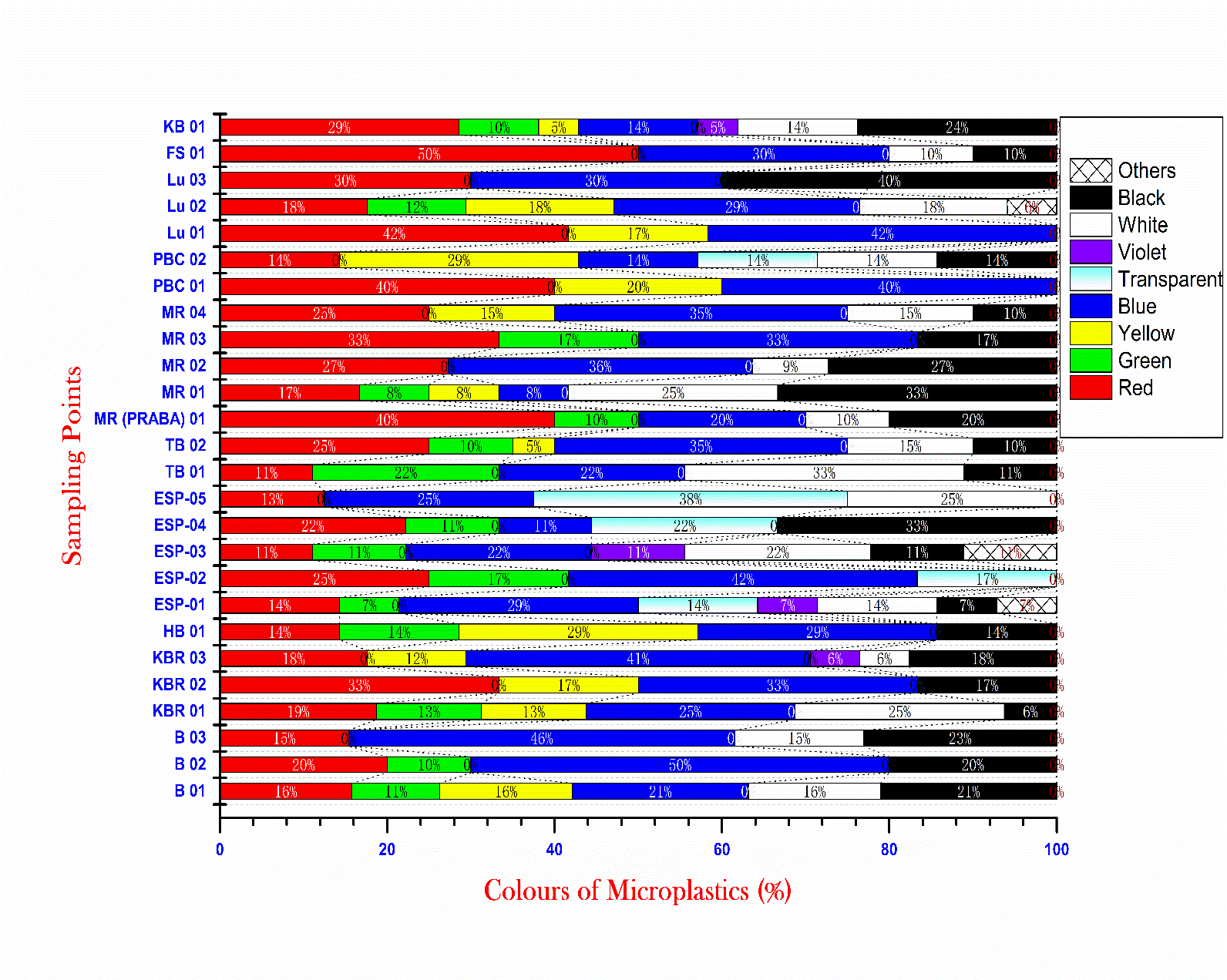


**Fig. 7** Colours of MPs determined in the Miri coast (in %) (Monsoon)


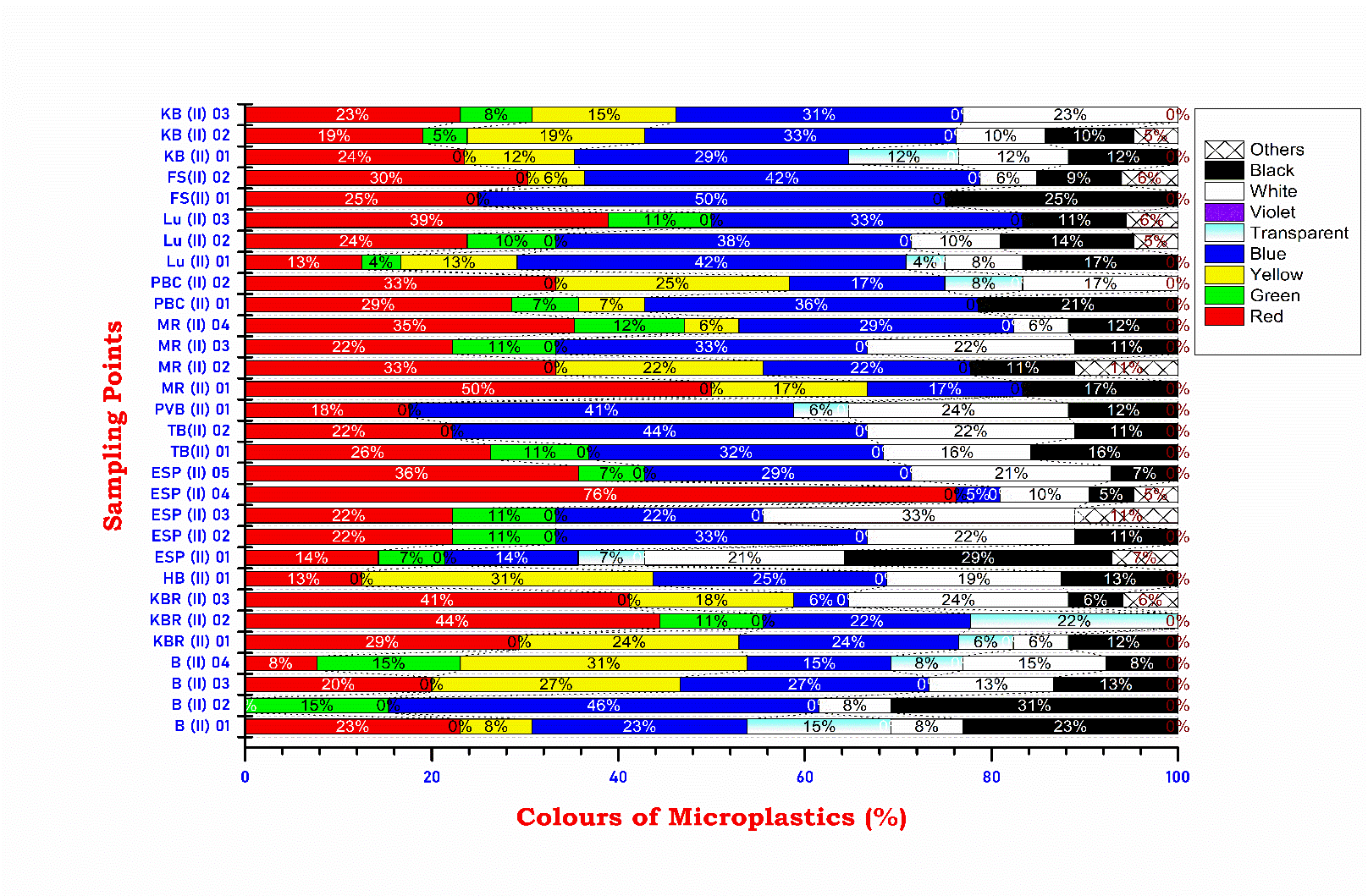


**Fig. 8** Colours of MPs identified in the Miri coast (in %) (Post-Monsoon)


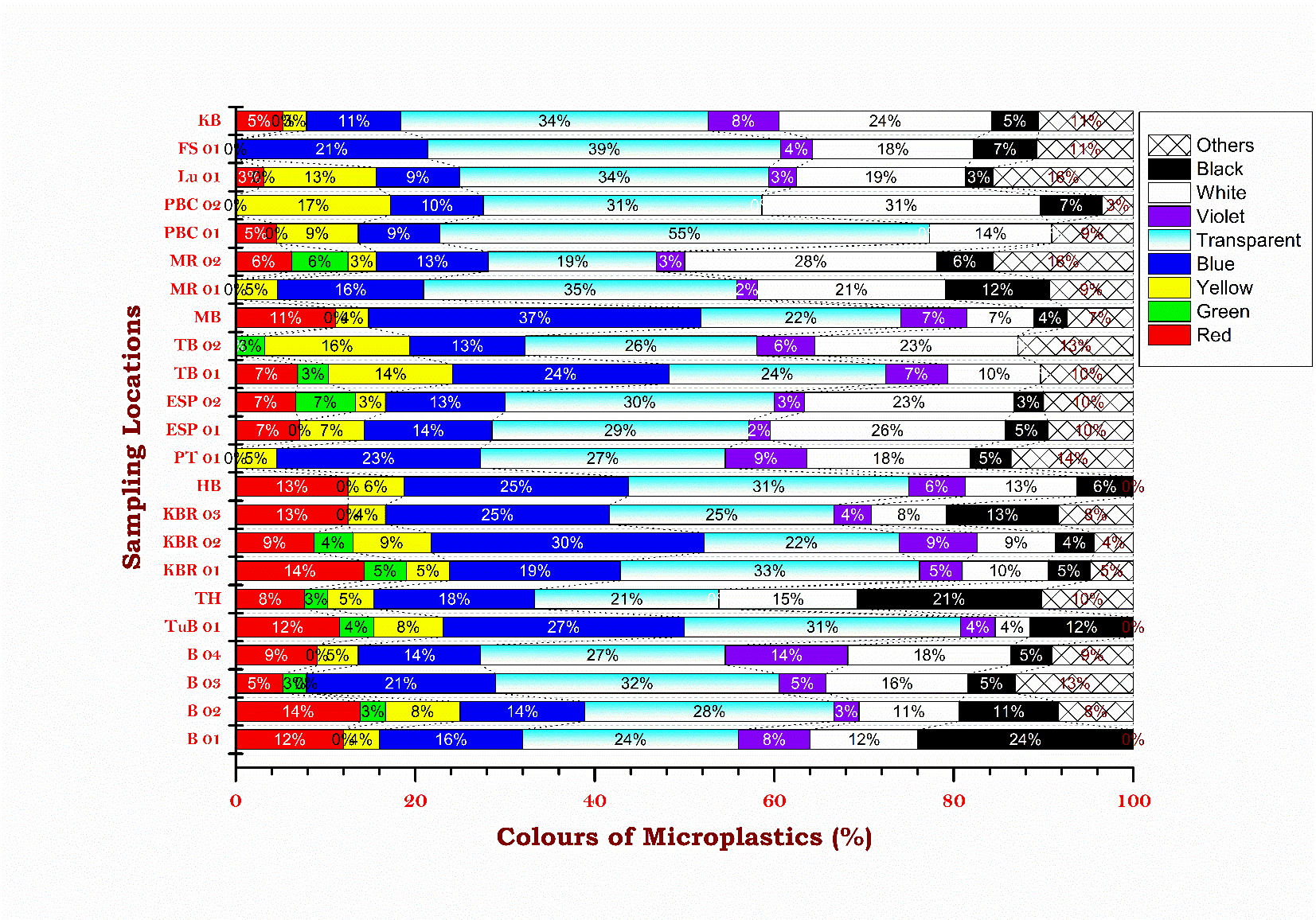


**Fig. 9** Identification of various colours of MPs in the Miri coast (in %) (Post-COVID)

**
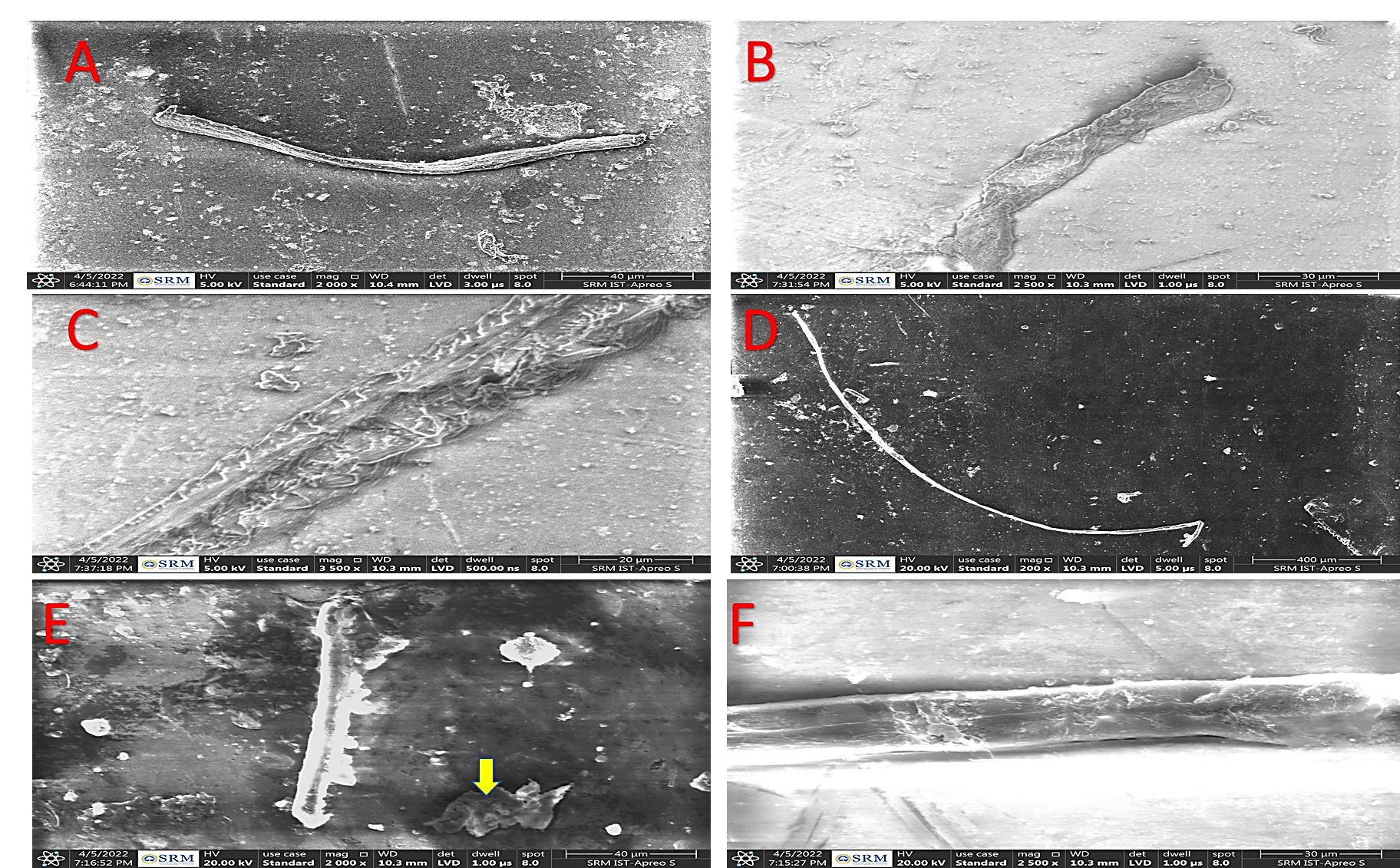
**

**Fig. 10** Microphotographs of MPs: A, D & F- Fibers; B- Film; C- Foam; E- Fragment (Shown by yellow arrow)

**
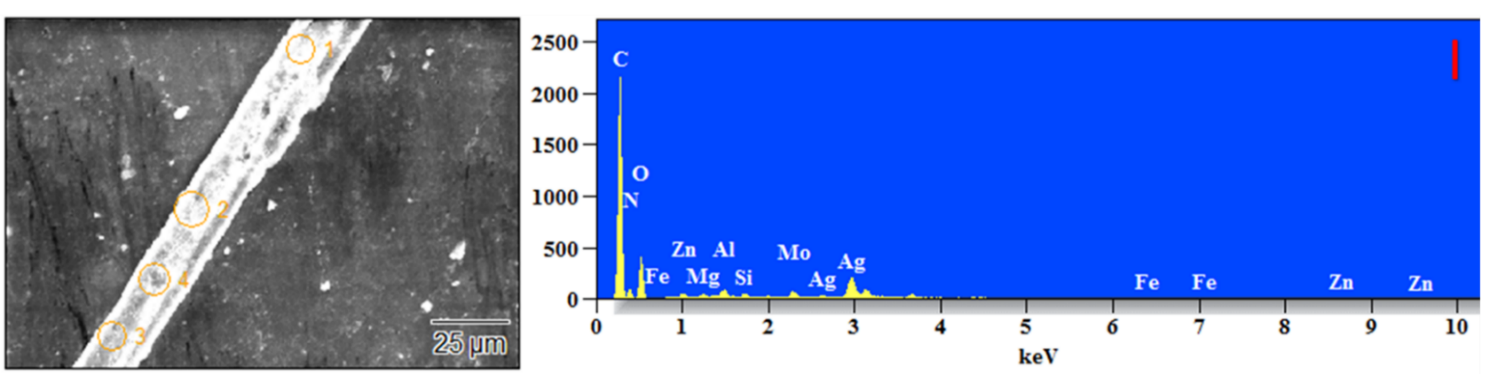
**
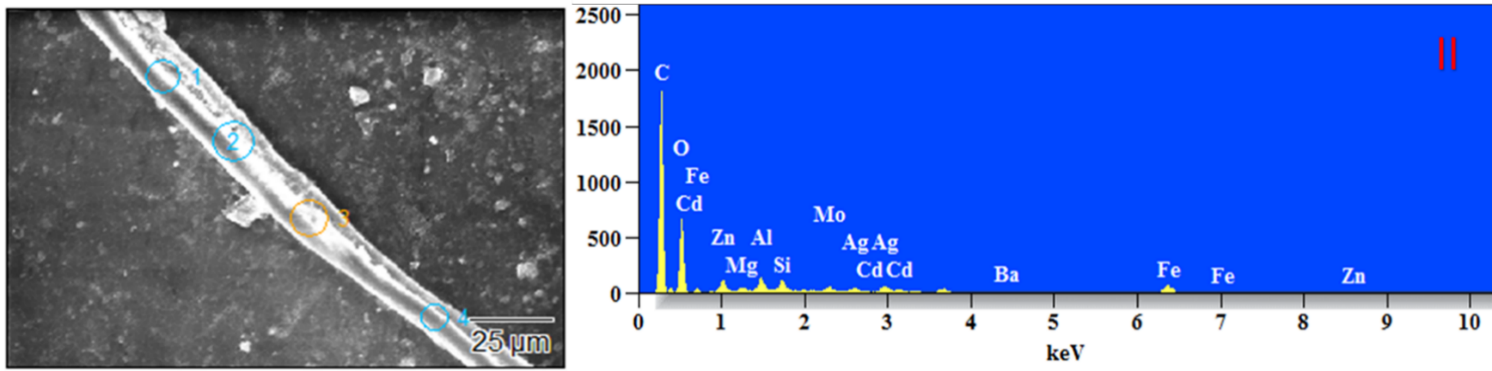


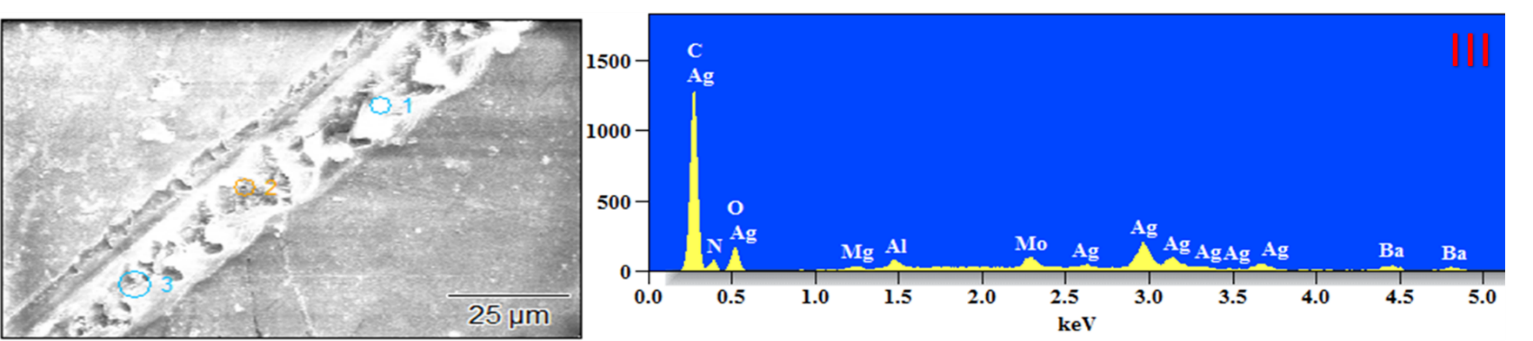

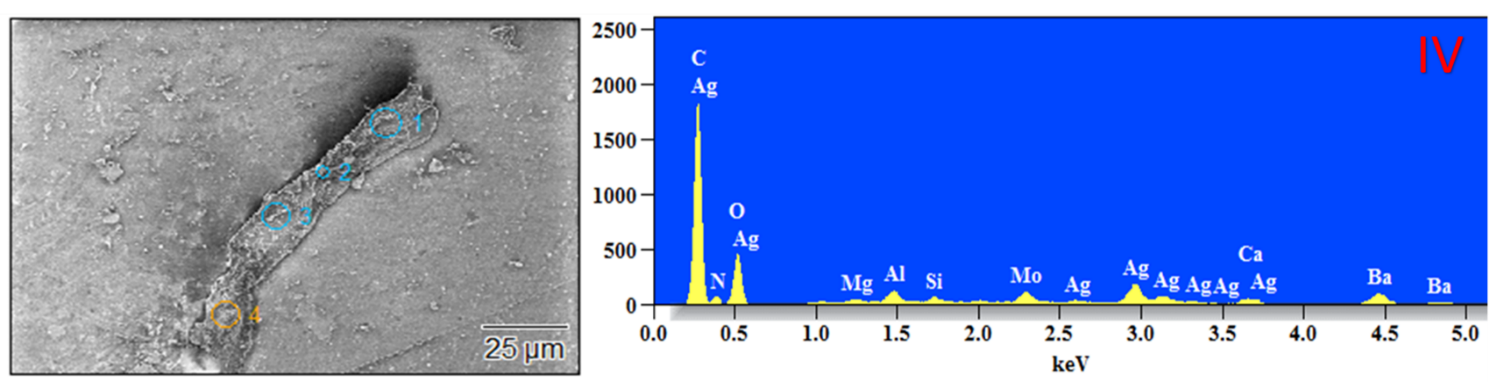


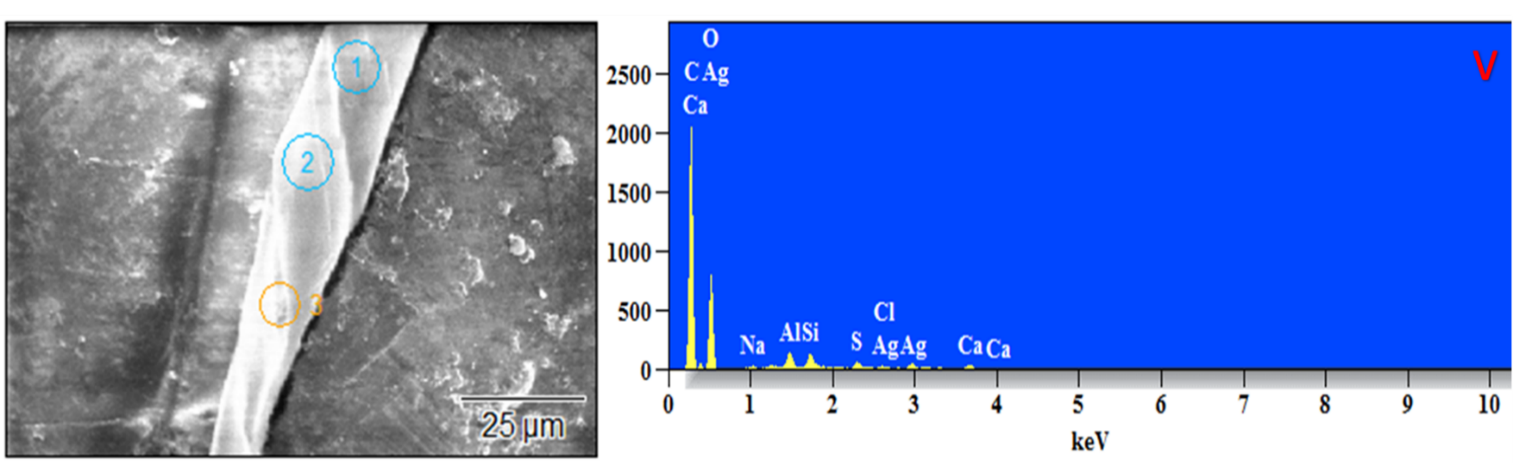


**Fig. 11** Microphotographs of MPs showing the morphologies of selected sites (1^st^ circular point view) accompanied by spectra of EDX: I, II & V- Fibers; III- Foam; IV- Film

**Table 2** Terminologies Illustrating the Level of Risk for MPs Pollution (R. Li et al., 2020; Ranjani et al., 2021)

| *PLI* | Hazard Category | *PHI* | Hazard Category | *PERI* | Risk Category |
| --- | --- | --- | --- | --- | --- |
| <10 | I | 0-1 | I | <150 | Minor |
| 10-20 | II | 1-10 | II | 150-300 | Medium |
| 20-30 | III | 10-100 | III | 300-600 | High |
| >30 | IV | 100-1000 | IV | 600-1200 | Danger |
|  |  | >1000 | V | >1200 | Extreme Danger |
